# Unstructured linker regions play a role in the differential splicing activities of paralogous RNA binding proteins PTBP1 and PTBP2

**DOI:** 10.1016/j.jbc.2024.105733

**Published:** 2024-02-08

**Authors:** Anthony Truong, Michael Barton, Uyenphuong Tran, Montana Mellody, Devon Berger, Dean Madory, Elizabeth Hitch, Basma Jibrael, Nikolas Nikolaidis, Tyler Luchko, Niroshika Keppetipola

**Affiliations:** 1 Department of Chemistry and Biochemistry, California State University Fullerton, Fullerton, California, USA; 2 Department of Physics and Astronomy, California State University, Northridge, Northridge, California, USA; 3 Department of Biological Sciences, California State University Fullerton, Fullerton, California, USA; 4 Department of Biological Science, Santa Ana College, Santa Ana, California, USA

**Keywords:** alternative splicing, RNA binding proteins, gene regulation, phosphorylation, molecular dynamics

## Abstract

RNA Binding Proteins regulate, in part, alternative pre-mRNA splicing and, in turn, gene expression patterns. Polypyrimidine tract binding proteins PTBP1 and PTBP2 are paralogous RNA binding proteins sharing 74% amino acid sequence identity. Both proteins contain four structured RNA-recognition motifs (RRMs) connected by linker regions and an N-terminal region. Despite their similarities, the paralogs have distinct tissue-specific expression patterns and can regulate discrete sets of target exons. How two highly structurally similar proteins can exert different splicing outcomes is not well understood. Previous studies revealed that PTBP2 is post-translationally phosphorylated in the unstructured N-terminal, Linker 1, and Linker 2 regions that share less sequence identity with PTBP1 signifying a role for these regions in dictating the paralog's distinct splicing activities. To this end, we conducted bioinformatics analysis to determine the evolutionary conservation of RRMs *versus* linker regions in PTBP1 and PTBP2 across species. To determine the role of PTBP2 unstructured regions in splicing activity, we created hybrid PTBP1-PTBP2 constructs that had counterpart PTBP1 regions swapped to an otherwise PTBP2 protein and assayed on differentially regulated exons. We also conducted molecular dynamics studies to investigate how negative charges introduced by phosphorylation in PTBP2 unstructured regions can alter their physical properties. Collectively, results from our studies reveal an important role for PTBP2 unstructured regions and suggest a role for phosphorylation in the differential splicing activities of the paralogs on certain regulated exons.

The process of alternative splicing allows one gene to code for more than one protein, increasing the protein diversity in higher eukaryotes. This process is regulated by *trans*-acting RNA binding proteins that bind sequence-specifically to *cis*-elements on the pre-mature gene transcript (1). Polypyrimidine Tract Binding Protein 1 (PTBP1) and its neuronal homolog, Polypyrimidine Tract Binding Protein 2 (PTBP2), are paralogous RNA binding proteins that function as splicing regulatory proteins to either promote or inhibit the assembly of the spliceosome at nearby splice sites (2, 3, 4). This action can impact the assembly of a functional spliceosome, influence splice site selection, and in turn the spliced isoforms generated from a gene transcript. PTBP1 is expressed near ubiquitously but is absent in neurons and myocytes, while PTBP2 is expressed primarily in neurons and testis (2, 5). The two proteins share 74% primary structure identity and share a similar domain arrangement of four RNA Recognition Motif (RRM) type RNA binding domains connected *via* three linker regions and an N-terminal region with nuclear import and export sequences (Fig. 1). The paralogs have near-identical RNA interacting residues except for three conservative changes where PTBP2 has two tyrosine to phenylalanine and one lysine to arginine substitution (Fig. 1*B*) (6). In turn, the paralogs display similar RNA binding across the transcriptome (7). PTBP1 and PTBP2 play a critical role in the process of neuronal development and maturation; neuronal progenitor cells express PTBP1; however, during differentiation, the level of PTBP1 protein goes down while that of PTBP2 goes up. This change in protein concentration alters the splicing of a set of neuronal exons that are critical for the development of axons, dendrites, and the formation of synapses (5, 8, 9, 10, 11). PTBP1 can compensate for the lack of PTBP2 in some developmental contexts and parts of the brain but not others, highlighting that paralogs have both redundant and distinct functions in neuronal pre-mRNA splicing (7). However, RNA binding does not correlate with PTBP1 and PTBP2 target specificity; exons that are differentially regulated by the paralogs exhibited similar binding by PTBP1 and PTBP2 (*i.e.*, difference in binding to distinctly regulated exons was not statistically significant) (7). This finding suggests the presence of additional features such as chemical modifications and cofactors that might influence their differential splicing activities.

**Figure 1:**
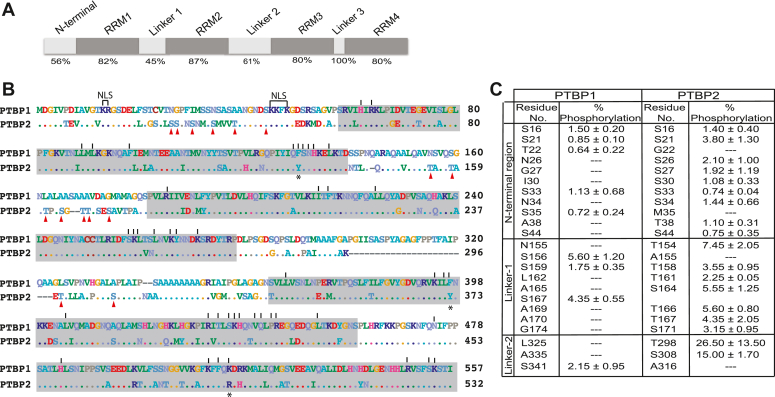
**PTBP1 and PTBP2 share less sequence identity and have distinct phosphorylated residues in the unstructured regions.***A*, schematic representation of the domain organization of PTB proteins. The percent amino acid sequence identity between human PTBP1 and human PTBP2 is indicated below each region. *B*, aligned amino acid sequences of PTBP1.4 (referred to as PTBP1 in this study) and PTBP2. *Dots* indicate residues that are identical between the paralogs. *Dashes* indicate deletions. *Vertical lines* above the PTBP1 sequence indicate residues that interact with the bound RNA per NMR solution structures (14). *Red triangles* below the PTBP2 sequence indicate PTBP2 distinct phosphorylated residues that do not have PTBP1 counterparts. NLS indicates the nuclear localization sequence. *Gray boxes* indicate the RNA binding domains. Star (∗) below the sequence indicate RNA binding residues that are not identical between the paralogs. *C*, residues that are phosphorylated in PTBP1 and PTBP2 under splicing reaction conditions containing HeLa nuclear extract described previously (12). Phosphate modifications in the N-terminal region, Linker 1 and Linker 2 regions are indicated for the paralogs.

We previously demonstrated that under splicing reaction conditions, PTBP1 and PTBP2 are post-translationally modified by the addition of phosphate and acetyl groups (12). Notably, phosphate modifications are localized to the N-terminal, Linker 1 and Linker 2 regions which are unstructured and share less sequence identity between the paralogs compared to the RNA binding domains (Fig. 1). Moreover, PTBP2 has many more distinct sites of phosphorylation and PTBP1 counterparts of these distinct modified residues are non-conservative substitutions with side chains that are unable to undergo phosphorylation (Fig. 1, *B* and *C*). Thus, it is plausible that the two proteins evolved to regulate distinct sets of exons by acquiring mutations encoding amino acids in divergent regions that can undergo phosphorylation. Moreover, PTBP1 Linker 1 and Linker 2 regions do not play a role in the splicing repression of certain neuronal exons including the N1 exon (13). Thus, we hypothesize PTBP2 linker regions contribute to the observed differences in splicing regulation.

Here, we aimed to test this hypothesis by probing sequence conservation, splicing activity, and structural properties of PTBP2 N-terminal, Linker 1 and Linker 2 regions. To address sequence conservation and to determine if the paralogs harbor conserved changes, we conducted a sequence alignment of PTBP1 and PTBP2 from a variety of vertebrate species. We created chimeric PTBP1-PTBP2 constructs by substituting PTBP1 N-terminal, Linker 1 and Linker 2 regions and combinations thereof into an otherwise PTBP2 protein. We assayed these chimeras for splicing activity with two exons that are differentially regulated by the paralogs. To evaluate how additional negative charges introduced by phosphate modifications might alter properties, including N/C-termini distance, radius of gyration, solvation volume, and secondary structure, we conducted molecular dynamics simulations of the isolated N-terminal, Linker 1 and Linker 2 regions.

## Results

### PTBP1 and PTBP2 sequence conservation

**Figure 2:**
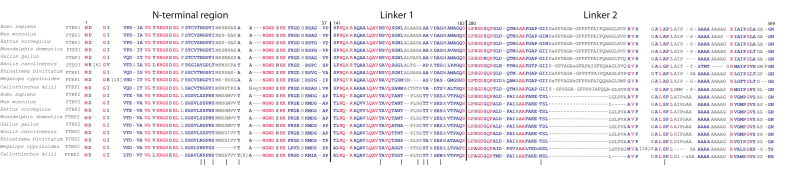
**Multiple sequence alignment of PTBP1 and PTBP2 regions reveals highly divergent unstructured regions.** Nine PTBP1 and nine PTBP2 protein sequences were collected from representative vertebrate species ranging from fishes to mammals using BLASTp. The protein sequences were aligned using the Constraint-based Multiple Alignment Tool (32) (COBALT). The amino acid residues were colored *red* if they were identical in all sequences used, *blue* if they were different, and *grey* if they belonged to regions with several large alignment gaps. For clarity, some large insertions-deletions (gaps) were removed and replaced with a number indicating the number of amino acids present in a particular protein sequence but absent from others. *Vertical lines* below the alignment indicate residues that are phosphorylated under splicing reaction conditions in PTBP2 (12).

A multiple sequence alignment of PTBP1 and PTBP2 from various species of jawed vertebrates highlights that the RRMs and the Linker 3 region are well conserved between the paralogs across species (Fig. S1). The Linker 3 region is structured and plays a critical role in creating a hydrophobic groove between RRMs 3 and 4 to orient them in a way that the RNA interacting β sheet surfaces are pointing away from the center (14, 15). This arrangement promotes each RRM to simultaneously bind both upstream and downstream PTBP1 binding sites of a regulated exon and can facilitate RNA looping, which has a role in a postulated mechanism of PTBP1-mediated splicing regulation (14, 16, 17). The N-terminal region, Linker 1 and Linker 2 regions are less well conserved between the paralogs (Fig. S1). We observed that many of the PTBP2 distinct phosphorylated residues in these regions (that did not have counterpart PTBP1 residues) are conserved changes (Fig. 2). This result signifies an important role for these residues (and phosphorylation) in PTBP2 function.

### PTBP2 unstructured regions play a role in cSrc N1 exon splicing regulation

**Figure 3:**
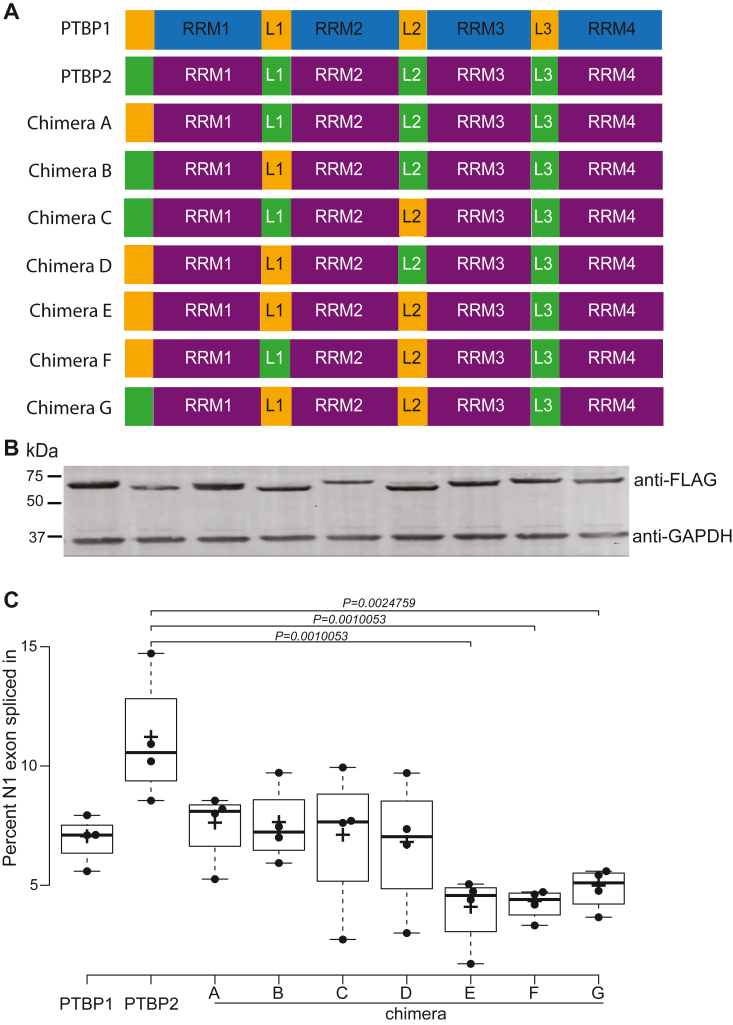
**PTBP2 unstructured regions play a role in N1 exon splicing regulation.***A*, schematic representation of chimeras constructed. PTBP1 RRMs and linker regions are indicated in *blue* and *yellow* respectively. PTBP2 RRMs and linker regions are indicated in *plum* and *green* respectively. Chimeric constructs follow similar color coding to indicate regions from PTBP1 and PTBP2. *B*, immunoblot of chimeric Flag-PTB proteins in cell lysates after transfection with 2.0 μg of Flag-PTBP plasmid DNA. Molecular weight standards are indicated on the *left*. Lanes are aligned with labeled x-axis lanes indicated in Panel *C*. *C*, PTBP1, PTBP2, and chimera splicing activity on the N1 exon. Splicing reporter Dup 4 to 5 (0.5 μg) was cotransfected with an empty expression vector (pcDNA 3.1+) or PTBP expression plasmid (2.0 μg). RNA was harvested after 48 h, assayed by RT-PCR, and quantified. The level of Percent-Spliced-In (PSI) was calculated by dividing the band intensity for the included product by the total value of excluded and included products. The PSI values (n = 4) were used to generate the boxplots using BoxPlotR code (58). *Center lines* show the medians; *box* limits indicate the 25th and 75th percentiles as determined by R software; whiskers extend 1.5 times the interquartile range from the 25th and 75th percentiles, outliers are represented by dots; crosses represent sample means; data points are plotted as *circles*. Statistical significance was determined by one-way ANOVA (Analysis Of Variance) with post hoc Tukey HSD (Honestly Significant Difference) test. Significant *p* values are presented above the plots.

Here, we aimed to determine whether PTBP2 unstructured regions play a role in its distinct splicing activity. To this end, we created a series of chimeric constructs containing PTBP1 unstructured regions in an otherwise PTBP2 protein *via* overlap extension PCR (Fig. 3*A*). The high degree of identity in domain organization and boundaries between the paralogs enabled us to create chimeras with exact changes in distinct regions. Our results indicate that all constructs are well expressed in mouse Neuro 2A cells (Fig. 3*B*). We assayed the proteins for splicing repression activity on the differentially regulated cSrc N1 exon (Fig. S3). PTBP1 represses the inclusion of the N1 exon in the final spliced mRNA while PTBP2 does so to a lesser extent (6, 18, 19, 20, 21). Our data highlight that introducing the PTBP1 N-terminal region (Chimera A), Linker 1 (Chimera B), Linker 2 (Chimera C) or a combination of the N-terminal and Linker 1 regions (Chimera D) in lieu of PTBP2 counterpart regions displayed splicing activity similar to wild type PTBP1 but did not alter PTBP2 splicing activity significantly (Fig. 3*C*). Thus, we surmise each unstructured region plays a role in PTBP2 splicing activity including a combination of the N-terminal and Linker 1 regions yet neither of these constructs (A, B, C, and D) could alter PTBP2 splicing activity significantly. In contrast, constructs that contained PTBP1 Linker 2 and a combination of either or both PTBP1 N-terminal and Linker 1 regions in an otherwise PTBP2 protein (Chimeras E, F, and G) demonstrated significantly different higher splicing repression activity (low percent spliced in) on the N1 exon compared to wild type PTBP2 (Fig. 3*C*). Thus, our data indicate an essential role played by PTBP2 Linker 2 region in its splicing activity as all chimeras that displayed a significant difference compared to wild-type PTBP2 contained the PTBP1 Linker 2 region. Our data also indicate an essential role for the presence of either the N-terminal or Linker 1 region (along with Linker 2) in PTBP2 distinct splicing activity. These findings suggest a cooperative behavior between Linker 2-Linker 1 or Linker 2-N terminal region in PTBP2 splicing regulation. Moreover, PTBP1 Linker 1 and Linker 2 regions are not required for splicing repression of the N1 exon; deletions of 40 out of 42 (Linker 1) and 70 out of 84 (for Linker 2) residues generated a mutant that could repress N1 exon splicing similar to wild-type PTBP1 (13). Collectively, our data support an essential role for the PTBP2 Linker 2 region and redundant roles for the N-terminal and Linker 1 region in dictating its distinct splicing regulation on the neuronal N1 exon. We note that PTBP2 Linker 2 region has two phosphorylation sites compared to the N-terminal and Linker 1 regions which have nine and seven respectively (Fig. 1).

### PTBP2 unstructured regions play a role in repressing the differentially regulated Dup175 text exon

We also assayed the chimeric constructs with the Dup175-DS9 minigene reporter that contains a 175-nucleotide test exon that is differentially regulated by the paralogs; PTBP1 represses inclusion of this test exon while PTBP2 does so to a lower extent (21, 22). We conducted co-transfections (chimera and reporter minigene) and carried out titrations for each chimera. Wild-type PTBP1 and PTBP2 served as controls (Fig. S2). Our data indicate that FLAG-construct expression is dependent on the concentration of plasmid; we observe DNA concentration-dependent protein expression. We harvested the RNA for each titration and carried out reverse-transcription PCR to assay for splicing changes of the Dup175 exon with increasing protein concentration. In agreement with previous studies, our data indicate that PTBP1 represses the inclusion of the Dup175 test exon and PTBP2 does so to a lesser extent (21, 22). We note that Chimera A repressed the Dup175 test exon to a similar level as PTBP2. Thus changes in the N-terminal region did not alter PTBP2 activity on the Dup 175 text exon. Chimeras B, C and D show repression trends that mimic PTBP1 similar to the pattern observed with the N1 exon. We surmise repression observed with Chimera D (N-terminal and Linker 1 region of PTBP1 in an otherwise PTBP2 protein) is exerted by the Linker 1 region. Thus, our data highlight Linker 1 and 2 regions play a role in PTBP2 splicing regulation of the Dup 175 test exon. Our data also reveal that Chimeras E, F, and G repress splicing of the test exon with values similar or greater than PTBP1 with increasing protein concentrations (Fig. S2 bottom panel). It is noteworthy that Chimera F (a combination of PTBP1 N-terminal and Linker 2 region) demonstrated higher repression values with increasing protein concentrations than Chimeras A and C indicating that a combination of the two regions can play a role and thus alter PTBP2 splicing activity. Overall, findings from this assay support and complement those with the neuronal N1 exon signifying a role for linker regions in dictating the different splicing activities of the PTBP paralogs.

### N-terminal and Linker 1 regions of PTBP2 are more compact and structured when unphosphorylated

Here, we sought to investigate the structural properties of PTBP2 N-terminal and Linker 1 regions *via* molecular dynamics simulations of the phosphorylated and unphosphorylated regions. Our data highlight PTBP2 N-terminal and Linker 1 regions show significant differences in N/C-termini distance, radius of gyration, partial molar volume, and secondary structure. Overall, the phosphorylated structures are more extended and have less α-helix content than the unphosphorylated structures.

The N-terminal and Linker 1 regions both have a longer N/C-termini distance and larger radius-of-gyration when phosphorylated (Table 1). The unphosphorylated Linker1 region of PTBP2 has a more compact conformation, with a radius of gyration of 12.11 ± 0.23 Å and N/C termini distance of 24.78 ± 1.02 Å compared to 19.11 ± 0.23 Å and 45.54 ± 0.99 Å when phosphorylated. Similarly, the unphosphorylated N-terminal region of PTBP2 has a radius of gyration of 13.85 ± 0.33 and N/C termini distance of 31.53 ± 1.25 compared to 25.02 ± 0.10 and 63.78 ± 0.47 when phosphorylated.

**Table 1:** Mean N/C termini distances, radii of gyration and partial molar volume for each sequence for all MD simulation copies

Sequence	Total number of residues	Net charge, e	N/C-termini distance, Å	Radius of gyration, Å	Partial molar volume, Å^3^
P1L1	42	0	19.35 ± 0.47	10.95 ± 0.04	4768 ± 4
P1phosL1	42	−6	14.01 ± 0.44	10.58 ± 0.06	4683 ± 4
P1L2	84	−2	19.73 ± 0.57	12.19 ± 0.04	9683 ± 8
P1phosL2	84	−4	20.25 ± 0.22	12.15 ± 0.04	9723 ± 7
P1Nterm	57	1	25.48 ± 0.56	11.90 ± 0.10	6852 ± 9
P1phosNterm	57	−9	22.16 ± 0.81	12.49 ± 0.16	6899 ± 10
P2L1	39	0	24.78 ± 1.02	12.11 ± 0.23	4713 ± 12
P2phosL1	39	−14	45.54 ± 0.99	19.11 ± 0.23	4614 ± 8
P2L2	62	−2	18.06 ± 0.86	11.12 ± 0.04	6983 ± 10
P2phosL2	62	−6	18.79 ± 0.4	11.42 ± 0.06	7052 ± 6
P2Nterm	57	−1	31.53 ± 1.25	13.85 ± 0.33	6961 ± 14
P2phosNterm	57	−19	63.78 ± 0.47	25.02 ± 0.10	6583 ± 4

Uncertainty is the standard error of the mean over the simulation copies.

**Figure 4:**
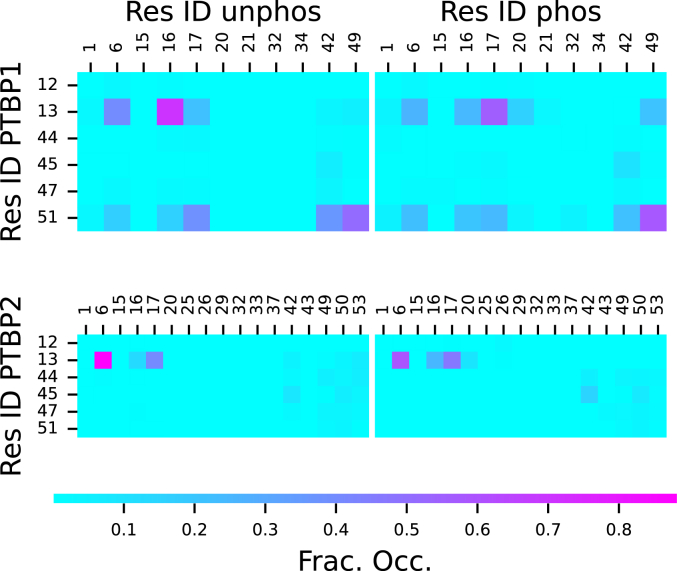
**Salt bridge fractional occupancies for N-terminal regions from MD simulations: (*top left*) P1Nterm, (*top right*) P1phosNterm, (*bottom left*) P2Nterm and (*bottom right*) P2phosNterm**.

**Figure 5:**
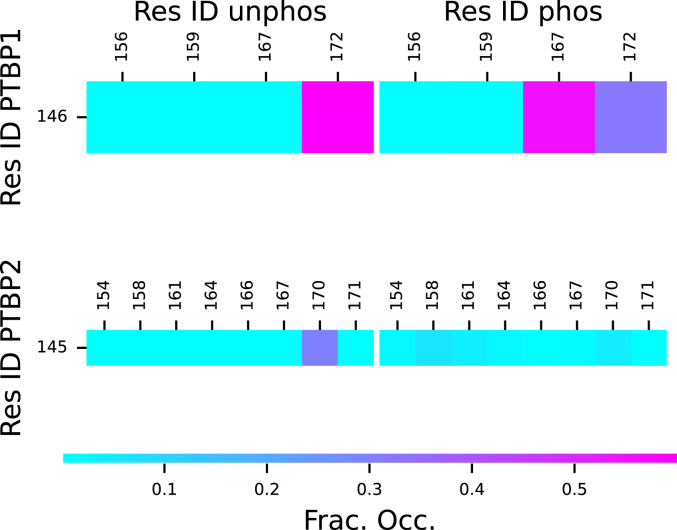
**Salt bridge fractional occupancies for Linker 1 regions from MD simulations: (*top left*) P1L1, (*top right*) P1phosL1, (*bottom left*) P2L1 and (*bottom right*) P2phosL1**.

As a result of this extension, both the N-terminal region and Linker 1 lose long-range (residues separated by more than five residues in the sequence) salt bridges when phosphorylated. Notably, residue 13 of P2phosNterm loses bridges with residues 42, 50, and 53 (Fig. 4). Other than low occupancy interactions that form between residues 12 and 26, and 13 and 20, all other new salt bridges are short-range (near neighbors in the sequence). P2phosL1 loses the only salt bridge in P2L1, residue 145 to 170 (Fig. 5). While this bridge only had occupancy of about 35%, it connected the mostly widely separated charged residues and helped reduce the N/C-termini distance. In contrast, residue 145 in P2phosL1 forms weak salt bridges with all possible partners at about 10% occupancy. This is likely due to random interactions in the rapidly shifting disordered structure.

**Figure 6:**
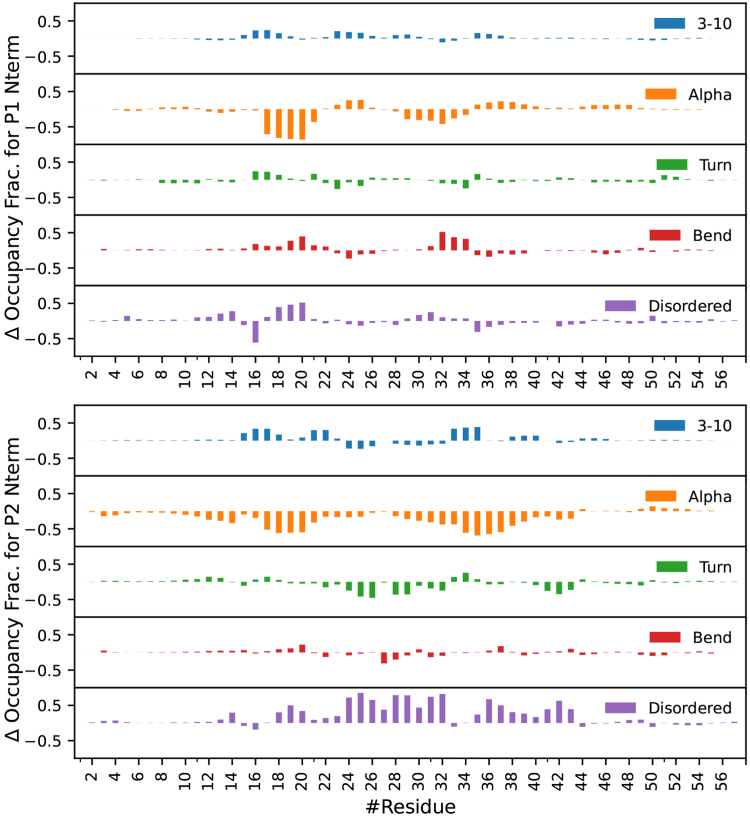
**Change in the secondary structure of the Nterm region after phosphorylation from MD simulations.** PTBP1 is shown at the *top*, PTBP2 is shown on the *bottom*. Total secondary structure of both sequences can be found in Figs. S4 and S6.

**Figure 7:**
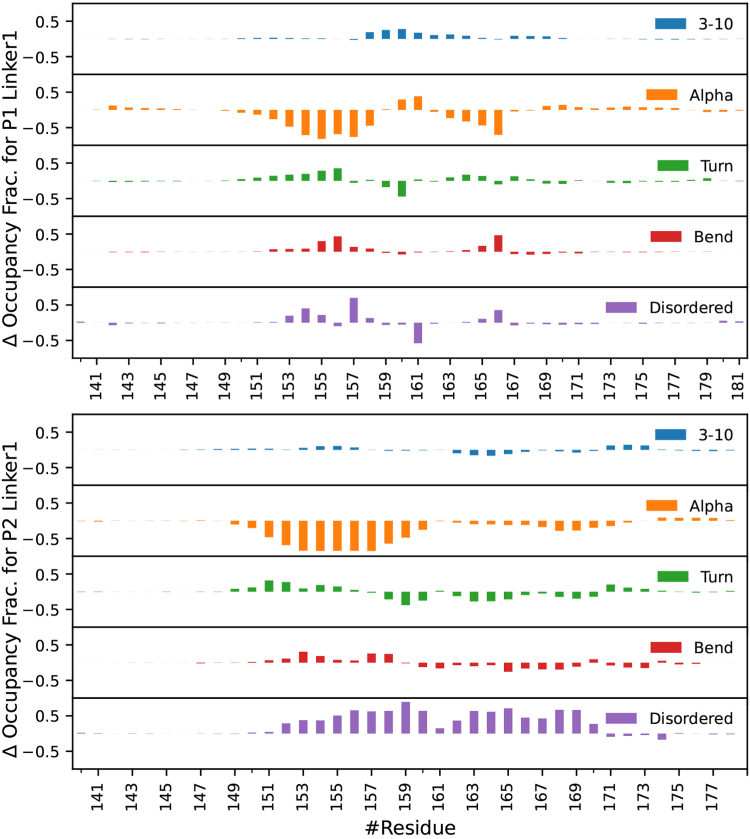
**Change in secondary structure of the Linker 1 region after phosphorylation from MD simulations.** PTBP1 is shown at the *top*, PTBP2 is shown on the *bottom*. Total secondary structure of both sequences can be found in Figs. S5 and S7.

The partial molar volume (PMV) is also reduced for both sequences when phosphorylated, as the PMV of P2Nterm is $6961\pm 14{Å}^{3}$ and P2L1 is $4713\pm 12\;{Å}^{3}$, while P2phosNterm is $6583\pm 4\;{Å}^{3}$ and P2phosL1 is $4614\pm 8\;{Å}^{3}$. This is the largest change in PMV among all sequences and is likely due to electrostriction, in which the increase in solvent-exposed charge draws the solvent closer to the solute and reduces the volume. There is a significant change in the net charge of both sequences, which are solvent-exposed (Table 1). Our results reveal that phosphorylation leads to a sharp decrease in the α-helix content of the PTBP2 N-terminal region (Figs. 6 and S4). This is most pronounced in residues 17 to 43, where α-helix content is almost completely extinguished and replaced by disordered structure. The disruption of the secondary structure and elongation of the peptide is due to the electrostatic repulsion of the phosphorylation sites, which introduce −18e charge into residue 16 to 44 (Table 2). We also observe a sharp reduction in α-helix content of the secondary structure for PTBP2 Linker 1 (Figs. 7 and S5). P2L1 is dominated by α-helix structure for residues 142 to 157, with weaker helical structure from residue 162 to 173. However, in P2phosL1 the stable α-helix begins to break down around residue 151 and is completely gone by residue 153, the neighbor of the first phosphorylation site at residue 154. The rest of the sequence is primarily disordered, with a small amount of α-helix and turn structure around the final phosphorylation site at residue 171. As with the N-terminal region, the introduction of large amount of charge (−14e) into a short sequence (residues 154–171) accounts for the absence of all helical structure.

**Table 2:** Phosphorylated residues for each region

Sequence	Phosphorylated residues
P1L1	S156, S159, S167
P1L2	S341
P1Nterm	S16, S21, T22, S33, S35
P2L1	T154, T158, T161, S164, T166, T167, S171
P2L2	T298, S308
P2Nterm	S16, S21, S26, S27, S30, S33, S34, T38, S44

### Phosphorylation causes shorter N/C-termini distances in PTBP1 N-terminal and linker 1 regions

Changes in N-terminal and Linker 1 regions in PTBP1 due to phosphorylation are smaller than for PTBP2 and suggest a more compact structure (Table 1). The radius of gyration for the P1L1 (10.95±0.04Å) was slightly larger than for P1phosL1 (10.58±0.06Å) and the average distance between the N/C termini of P1L1 (19.35±0.47Å) was significantly larger than for P1phosL1 (14.01±0.44Å). In contrast, the radius of gyration for the P1Nterm (11.90±0.1Å) was slightly smaller than for P1phosNterm (12.49±0.06Å), while the average distance between the N/C termini of P1Nterm (25.48±0.56Å) was significantly larger than for P1phosNterm (22.16 ± 0.81 Å). In both cases, there is only a small decrease in the PMV (<2%) when phosphorylation is introduced. Phosphorylation disrupts the α-helix secondary structure of both the N-terminal and Linker 1 regions. However, because there are many fewer phosphorylation sites than for the same region in PTBP2, the decrease in α-helix content is smaller. P1Nterm has significant α-helix content (Fig. S6), with hinges at residues 16 and 27. This α-helix content is lost is between residues 17 to 21, which lie between the phosphorylated sites 16 and 21 to 22 (Fig. 6). Similarly, α-helix structure is completely lost between residue 31 and 34, which are adjacent to phosphorylation sites at residues 33 and 35.

The Linker 1 region of PTBP1 has especially high α-helix content throughout the almost the entire sequence (Fig. S7). The phosphorylation of residues 156 and 159 (Table 2) greatly diminishes the α-helix content between residues 153 and 158 (Fig. 7). When phosphorylated, residue 167 causes a small disruption in the α-helix by forming a new salt bridge with residue 146 (Fig. 5).

### Linker 2 shows no changes due to phosphorylation in PTBP1 and PTBP2

**Figure 8:**
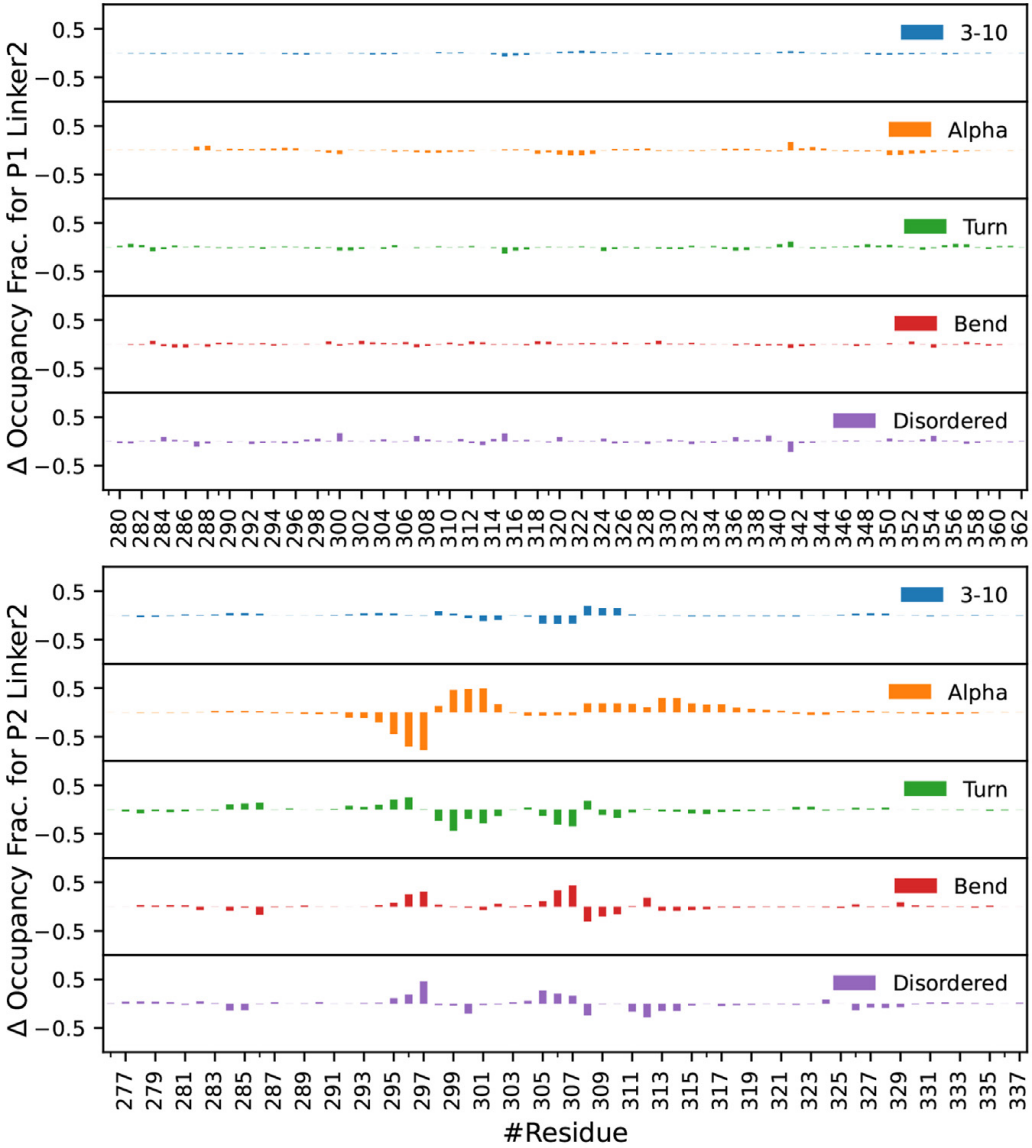
**Change in secondary structure of the Linker 2 region after phosphorylation from MD simulations.** PTBP1 is shown on *top*, PTBP2 is shown on the *bottom*. Total secondary structure of both sequences can be found in Figs. S8 and S9.

There was no significant change in the N/C-termini distance, radius of gyration, PMV or secondary structure for the Linker 2 region of either PTBP1 or PTBP2 due to phosphorylation (Table 1 and Fig. 8). Though the tertiary structure of both linker peptides was observed to be disordered, the secondary structure of both PTBP1 and PTBP2 contained several regions with significant α-helix content (Figs. S8 and S9). Notably, PTBP2 had two regions with significant helical content, residues 288 to 298 and 315 to 324, which correspond to conserved regions on PTBP1 with similar helical content, residues 289 to 297 and 342 to 354. The additional residues in PTBP1 formed short helical sections.

**Figure 9:**
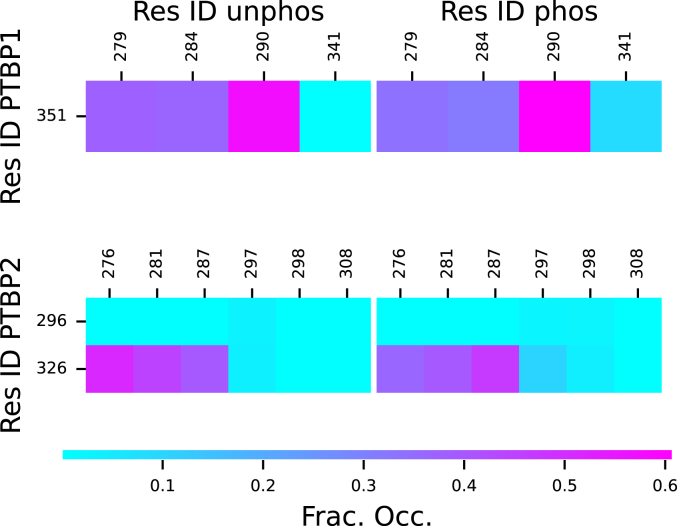
**Salt bridge fractional occupancies for Linker 2 regions from MD simulations: (*top left*) P1L2, (*top right*) P1phosL2, (*bottom left*) P2L2 and (*bottom right*) P2phosL2**.

While there were no large-scale changes in the structure of PTBP2 Linker 2 due to phosphorylation, there were small changes to the secondary structure near the phosphorylation sites. Notably, there was a decrease in α-helix for residues 294 to 297 and an increase for residues 298 to 302 (Fig. S9). This change was likely due to residue T298 forming low occupancy salt-bridges with K296 and R326 (Fig. 9), which also appears to stabilize a salt bridge between R326 and E297. These low occupancy salt bridges appear to have disrupted the α-helix at 295 to 297 and allowed the new α-helices to form at residues 298 to 302 and 308 to 312. Conversely, PTBP1 Linker 2 showed no significant change in secondary structure due to phosphorylation, and the only change to the observed salt bridges was a new low occupancy bridge between residues 341 and 351.

## Discussion

We found that the unstructured N-terminal, Linker 1 and Linker 2 regions of PTBP2 regulate its splicing activity on certain exons including the neuronal N1 exon. Our data indicate an important role played by PTBP2 Linker 2 region in combination with either the N-terminal or Linker 1 region in exerting is distinct splicing activity. PTBP2 is phosphorylated at 9, 7, and 2 positions in the N-terminal, Linker 1 and Linker 2 regions, respectively. Phosphorylated residues are conserved in lower species indicating an important role in function. Due to the difference in the length of the Linker 2 region in PTBP1 (84 aa) *versus* PTBP2 (62 aa), PTBP2 Linker 2 has a significantly smaller PMV compared to PTBP1 Linker2. The Linker 2 region was the only unstructured region that did not show significant large-scale structural differences when phosphorylated in both PTBP1 and PTBP2. Previous work highlights the length of the Linker 2 region in PTBP1 does not play a role in regulating N1 exon splicing (13). Thus, we conclude the regulatory effects of Linker 2 are not due to changes in the large-scale physical properties of the linker, such as the structure or net charge. We surmise that the Linker 2 region contributes to PTBP2 splicing activity through mediating inter-RRM interactions, PTBP2-RNA and/or protein-protein interactions, rather than through the mechanical behavior of the linkers. One possible mechanism is the change in PTBP2 Linker 2 region secondary structure α-helical content with phosphorylation at Thr298 and Ser308 while changes in PTBP1 are minimal. These changes in secondary structure may affect how Linker 2 interacts with other regions or molecules and will be addressed in future work.

Our molecular dynamics simulations results indicate that the density of phosphorylated residues correlates strongly with physical changes in the sequences. P2phosNterm and P2phosL1 show the largest changes and have the largest number of total phosphorylated residues (9 and 7) and fraction of phosphorylated residues (0.158 and 0.179) (Tables 1 and 2). Conversely, P1phosL2 and P2phosL2 are the longest sequences, have lowest number of phosphorylated sites (1 and 2) and lowest fraction of phosphorylated residues (0.012 and 0.032). This also explains why P2L1 showed large conformational changes when phosphorylated while P1phosNterm and P1phosL1 did not. Furthermore, the greatest disruptions to secondary structure occurred where the phosphorylated residues were most closely spaced. For P2phosNterm and P2phosL1, Coulomb repulsion between the phosphorylated residues led to structures that were less ordered and more extended compared to P2Nterm and P2L1. A possible physical mechanism is that the phosphorylation of these sequences causes more separation between the RRM domains and influence RNA binding and/or protein-protein interactions required for PTBP2 splicing activity.

A possible limitation of the simulations is the use of the Onufriev-Bashford-Case-2 (OBC2) generalized Born (GB) implicit solvent model (23), with the Amber FF19SB force field (24). We used temperature replica exchange molecular dynamics (TREMD), which has been shown to capture conformational changes in IDPs, such as those we observed (25). However, TREMD would be infeasible using explicit solvent models due to the large number of degrees of freedom. Therefore, we use the GB solvent model. While the use of GB with earlier Amber force fields is known to have a slight helical bias (25), improvements to the helical propensity have been made for Amber FF19SB (24). While unphosphorylated P1L1 and P2L1 show significant helical content, the other peptides are disordered, as expected. When phosphorylated, P1L1 and P2L1 lose most of their helical content, which is consistent with our overall finding that the structure strongly depends on the net charge of the peptide. Future studies will aim to determine the role of phosphate modifications in the N-terminal and Linker 1 region on PTBP2 function. In summary, our studies highlight that the less conserved unstructured regions between paralogs PTBP1 and PTBP2 play a role in their distinct splicing activities. PTBP2 has conserved changes in these regions including residues that have side chain hydroxyl groups that can undergo reversible phosphorylation. Our work also suggests a role for phosphate modifications in these unstructured regions to contribute to the neuronal-specific splicing activity of PTBP2.

## Experimental procedures

### Cell culture and transfections

Mouse neuro 2A (N2A) cells were grown according to the American Type Culture Collection (ATCC) recommended protocols in DMEM with 10% FBS (Omega scientific) and 1× PenStrep Glutamine. Transfections were carried out using wild-type cells with Lipofectamine 3000 transfection reagent (Life Technologies) according to the manufacturer’s instructions to overexpress FLAG-PTBP and FLAG-Chimeric constructs. Cells were harvested 48 h post transfection and RNA and protein were isolated for analysis.

### Plasmid construction

Chimeric constructs were created by 2-step over-lap extension PCR. Primers were designed to amplify regions of PTBP1 or PTBP2, carrying overlapping flanking sequences (Table S2), and purchased from Integrated DNA Technologies. The first PCR reactions generated fragments using either PTBP1 or PTBP2 as template. Reaction mixtures (50 μl) contained 0.2 mM dNTPs, 1 uM forward and reverse primers, 1× Phusion Buffer, 30 ng of template DNA and 0.5 ul of Phusion Plus DNA polymerase. The second PCR reaction combined fragments for each chimera (generated in the first PCR reactions) to generate the full-length chimeric gene with primers containing BamH1 and EcoRV cut sites at the 5′ and 3′ ends respectively. The PCR fragments were cloned into pcDNA3.1(+) (Life Technologies) using its BamH1 and EcoRV restriction sites and T4 DNA ligase. The inserts were sequenced to verify the absence of unwanted coding changes during PCR amplification. Each expression plasmid carried an N-terminal FLAG-tag. The minigene reporter Dup 175-DS9 contained a 175-nt hybrid exon obtained from joining the 5′ end of β globin exon2 to the 3′ end of β globin exon1 (26, 27). The minigene reporter contains an exonic and upstream intronic PTBP binding site from c-Src. The test exon is flanked by wild–type β globin exons 1 and 2 (22). The minigene Dup 4 to 5 contains the Src N1 exon with upstream and downstream intronic regulatory regions inserted between the flanking β globin exons of Dup175 (19, 28, 29).

### Reverse-transcription PCR

RNA was isolated from N2A cells using the PureLink RNA Mini Kit (Invitrogen) and reverse transcribed with random hexamers and Superscript III (Life Technologies) according to the manufacturer’s instructions. Spliced products were PCR amplified (22 cycles) using a 5′ primer (DUP-8 5′-GACACCATGCATGGTGCACCTG-3′) and AlexaFluor 488 (Thermo Fisher) labeled 3′ primer (DUP-3) 5′-AACAGCATCAGGAGTGGACAGATCCC-3′ (30). The PCR products were separated by 8% Acrylamide/7.5 M Urea denaturing gels and visualized by a Typhoon FLA7000 PhosphorImager. Bands corresponding to the length of included and excluded products were boxed and band intensity was quantified using ImageQuant TL software. Percent spliced-in values were calculated by dividing the band intensity of the included product by the total intensity of included and excluded products and multiplying by 100.

Statistical significance was determined by one-way ANOVA with post hoc Tukey HSD (Honestly Significant Difference) test. A *p* value <0.05 was considered statistically significant. The boxplots were generated using R software (http://shiny.chemgrid.org/boxplotr/).

### Immunoblotting

Whole-cell lysates were separated by 12% Acrylamide SDS-PAGE, transferred to an Immobilon PVDF membrane, and probed with anti-Flag (Sigma catalog# F3165) and GAPDH antibodies (Invitrogen 6C5 *via* Fisher Scientific) at 1:2500 dilutions. After incubation with fluorescent-conjugated Alexa488 secondary antibody (Invitrogen A11001 *via* Fisher Scientific) at 1:2500 dilution, the blots were scanned using a Typhoon FLA7000 PhosphorImager.

### Multiple sequence alignment

Nine PTBP1 and nine PTBP2 protein sequences were collected from representative vertebrate species ranging from fishes to mammals. These sequences were identified using BLASTp (31) and the human protein sequences as queries. The database used was the ref_prot (containing only reference protein sequences from well-annotated genomes) and the search was performed using default parameters. The protein sequences were collected and aligned using the Constraint-based Multiple Alignment Tool (COBALT) (32). The amino acid residues were color-coded based on the identity of a particular multiple sequence alignment color. Specifically, they were colored red if they were identical in all sequences used, blue if they differed even in one amino acid, and gray if they belonged to regions with several large alignment gaps. For clarity, some large insertions-deletions (gaps) were removed and replaced with a number indicating the number of amino acids present in a particular protein sequence but absent from others.

### Molecular dynamics simulation methods

#### Model preparation A

Starting structures in extended conformation were generated with leap (24) for PTBP1 and PTPB2 wild-type and phosphorylated sequences. Phosphorylated residues for each region are detailed in Table 2. The most common protonation states for pH 7 were assigned to all titratable residues; histidine was protonated at the N ϵ only. The N and C termini of each sequence were capped with neutral acetyl (ACE) and N-methylamide (NME) groups to avoid introducing charged groups. The Amber FF19SB (33) forcefield was used along with associated phosphorylated amino acid parameters from phosaa19SB. Masses of hydrogen atoms and their associated heavy atoms were repartitioned with parmed to allow a 4 fs time step in the molecular dynamics calculations (30, 34). To relax the initial structures, 1000 steps of limited memory Broyden-Fletcher-Goldfarb-Shanno energy minimization were carried out with the XMIN module of sander using default cutoffs (35, 23).

For all energy minimization and molecular dynamics calculations, the generalized Born (GB) implicit solvent model was used with the OBC2 model, igb = 5, and Born radii set to the recommended mbondi2 parameters (36). A 150 mM concentration of ions was modeled using the modified generalized Born theory based on the Debye-Hückel limiting law for ion screening of interactions (37). In Amber suite, the non-polar contribution to the non-polar hydration free energy is modeled with a simple surface area term, which is known to be inaccurate (38, 39). As this term had relatively high computational cost and known problems, we did not use the non-polar contribution of the GBSA model was not used, (gbsa = 0) (38).

#### Temperature replica exchange MD (TREMD)

TREMD simulations (40) were used to sample conformations of the peptides with a temperature range of 275 K to 500 K (Table S1). Temperatures were selected to achieve a target exchange probability of 0.4, using the virtualchemistry.org REMD temperature generator, with the number of atoms taken from the phosphorylated systems (41) (Table 1). The same temperatures were used for both phosphorylated and unphosphorylated systems. Exchanges were attempted every time step (42) and an average exchange rate of ∼0.33 was observed.

For each of the structures in this study, five independent copies were simulated using TREMD with different random seeds, yielding different initial velocities and random number sequences for Langevin dynamics. Hydrogen bond lengths were constrained with SHAKE (43), which allowed a time step of 4 fs due to hydrogen mass repartitioning. Temperature was controlled with Langevin dynamics using default parameters (44). Long-range cutoffs were not used for non-bond interactions. The simulations were run until the standard error of the N/C-termini distance and radius-of-gyration across all five TREMD simulations was <5%. This stopping condition ensured an extended period of reduction in the standard error, consistent with convergence (45). In all, MD simulations were run out to 300 ns, and the first 50 ns were discarded. One out of every 2500 frames of the trajectory was output for analysis, resulting in 25,000 frames for each independent copy.

#### Simulation analysis

Before analysis, trajectories for the target temperature of 298 K were assembled for each TREMD simulation using cpptraj (46).

Cpptraj was used to calculate the radius of gyration, N/C-termini distance, secondary structure, and net charge. The net charge is defined as the sum of all solute charges, which also can be calculated as the sum of the basic (+e) and acidic (−e) amino acids, and phosphate (−2e) groups.

Salt bridge interactions were determined using an in-house script. For each frame of a simulation trajectory, a salt bridge interaction was counted as occupied when two oppositely charged residues came within 4 Å (47, 48, 49) of one another. As the net charge is localized at the ends of the side chains, only ASP O δ, GLU O ϵ, ARG N η, LYS N ζ, and the TPO and SEP phosphate oxygen atoms were considered when calculating distances. Salt bridge fractional occupancies for each simulation trajectory are shown in Figure 4, Figure 5 and 9.

#### Partial molar volumes

Partial molar volumes (PMVs) were calculated using 3D-RISM in AmberTools 22 (50, 51) for 1000 frames randomly chosen from the trajectories. 3D-RISM requires the bulk properties of the solvent, which we calculated with the rism1d program, following the procedures of Joung *et al.* (52). The coincident extended simple point charge model (cSPC/E) (51) was used for water, with a concentration of 150 mM, and Joung-Cheatham parameters for sodium chloride ions (53) were used, with a concentration of 0.15 M. The dielectrically consistent RISM equation (54) was solved on a radial grid of 16,384 points at a temperature of 298 K with a dielectric constant of 78.446 to a residual tolerance of $10^{-12}$ using partial series expansion of order-3 (PSE-3) closure (55). The solution was accelerated using the modified direct inversion of the iterative subspace (MDIIS) solver (56).

Using the bulk solvent properties from rism1d, the rism3d.snglpnt program was used to solve 3D-RISM with the PSE-3 closure to a residue tolerance of $10^{-4}$, accelerated by MDIIS. The 3D-RISM equations were solved on a grid with 0.4 Å spacing and the physical dimensions were set to ensure a minimum buffer distance of 24 Å between the solute and the edge of the grid. Open boundary conditions were employed, with analytically corrected Lennard-Jones-potential-energy cutoff of $10^{-5}$, reciprocal-space-asympotics cutoff of $10^{5}$, and treecode summation for real-space Coulomb and long-range-asymptotics interactions using a multipole acceptance criterion of 0.3 and fourth-order Taylor expansion (57).All 3D-RISM calculations converged except for one frame of P1phosNterm.

## Data availability

All data described are located within the manuscript.

## Supporting information

This article contains supporting information (32).

## Conflict of interest

The authors declare that they have no known competing financial interests or personal relationships that could have appeared to influence the work reported in this paper.

## Supporting information

Supporting Figure S1 Nine PTBP1 and nine PTBP2 protein sequences were collected from representative vertebrate species ranging from fishes to mammals using BLASTp. The protein sequences were aligned using the Constraint-based Multiple Alignment Tool (32). The amino acid residues were colored red if they were identical in all sequences used, blue if they were different, and grey if they belonged to regions with several large alignment gaps. For clarity, some large insertions-deletions (gaps) were removed and replaced with a number indicating the number of amino acids present in a particular protein sequence but absent from others. The accession numbers of the sequences used are *Homo sapiens* (PTBP1) (NP_002810.1); *Mus musculus* (PTBP1 (NP_001070831.1); *Rattus norvegicus* (PTBP1) (NP_001257986.1); *Monodelphis domestica* (PTBP1) (XP_001375584.2); *Gallus gallus* (PTBP1) (XP_015155407.1). *Anolis carolinensis* (PTBP1) (XP_008101639.2); *Rhinatrema bivittatum* (PTBP1) (XP_029469360.1); *Megalops cyprinoides* (PTBP1) (XP_036373891.1). *Callorhinchus milii* (PTBP1) (XP_007899197.1); *Homo sapiens* (PTBP2) (NP_001287914.1); *Mus musculus* (PTBP2) (NP_062423.1); *Rattus norvegicus* (PTBP2) (XP_006233298.1); *Monodelphis domestica* (PTBP2) (XP_007485072.1); *Gallus gallus* (PTBP2) (XP_004936631.4); *Anolis carolinensis* (PTBP2) (XP_008107427.1); *Rhinatrema bivittatum* (PTBP2) (XP_029474787.1); *Megalops cyprinoides* (PTBP2) (XP_036409945.1); *Callorhinchus milii* (PTBP2) (XP_042191263.1).

Supporting Figure S2 PTBP1, PTBP2 and chimera splicing activity on the Dup175 test exon. Each panel includes an immunoblot (top) and UREA-PAGE gel image (bottom) of protein expression and spliced products respectively of titrations of wild type and chimeric PTBP constructs. Immunoblots contain Flag-PTB proteins in cell lysates after transfection with increasing concentration of Flag-PTBP plasmid DNA (0.25, 0.5, 1.0, 1.5 and 2.0 ug). Molecular weight markers are indicated to the left of the first immunoblot in each panel. Immunoblots for PTBP2, Chimeras C, F and G do not contain the empty vector control sample lane. Splicing reporter minigene Dup 175-DS9 (0.5 μg) was cotransfected at each DNA concentration. RNA was harvested after 48 h, assayed by reverse transcription-PCR, and quantified. The level of percent-spliced-out was calculated by dividing band intensity for the excluded product by the total value of excluded and included products in each lane. The level of percent spliced out at each DNA concentration (0.25, 0.5, 1.0, 1.5, 2.0 ug) is calculated and indicated below each lane. Std.Deviations for wild type PTB proteins and mean error for chimeric constructs are indicated.

Supporting Figure S3 PTBP1, PTBP2 and chimera splicing activity on the neuronal N1 exon. Splicing reporter minigene Dup 4 to 5 (0.5 μg) was cotransfected with each FLAG construct into N2A cells. RNA was harvested after 48 h, assayed by reverse transcription-PCR using a fluorescent labeled reverse primer (5Alex488N/AACAGCATCAGGAGTGGACAGATCCC) and separated on a 7.5 M Urea 8% Acrylamide gel. N1 exon included and excluded band intensities were quantified using Image quant software. The level of percent-spliced-in was calculated by dividing band intensity for the included product by the total value of excluded and included products in each lane.

Supporting Figure S4 Secondary structure fractional occupancy of PTBP2’s N-terminal region from MD simulations: (top) P2Nterm, (bottom) P2phosNterm.

Supporting Figure S5 Secondary structure fractional occupancy of PTBP2’s Linker 1 region from MD simulations: (top) P2L1, (bottom) P2phosL1.

Supporting Figure S6 Secondary structure fractional occupancy of PTBP1’s N-terminal region from MD simulations: (top) P1Nterm, (bottom) P1phosNterm.

Supporting Figure S7 Secondary structure fractional occupancy of PTBP1’s Linker 1 region from MD simulations: (top) P1L1, (bottom) P1phosL1.

Supporting Figure S8 Secondary structure fractional occupancy of PTBP1’s Linker 2 region from MD simulations: (top) P1L2, (bottom) P1phosL2.

Supporting Figure S9 Secondary structure fractional occupancy of PTBP2’s Linker 2 region from MD simulations: (top) P2L2, (bottom) P2phosL2.
