# Supplementary material for: Unstructured linker regions play a role in the differential splicing activities of paralogous RNA binding proteins PTBP1 and PTBP2
Source: J Biol Chem. 2024 Feb 8;300(3):105733. doi: 10.1016/j.jbc.2024.105733 (PMC10914480; doi:10.1016/j.jbc.2024.105733)
Supplement: Supporting Figure S1 — Nine PTBP1 and nine PTBP2 protein sequences were collected from representative vertebrate species ranging from fishes to mammals using BLASTp. The protein sequences were aligned using the Constraint-based Multiple Alignment Tool (32). The amino acid residues were colored red if they were identical in all sequences used, blue if they were different, and grey if they belonged to regions with several large alignment gaps. For clarity, some large insertions-deletions (gaps) were removed and replaced with a number indicating the number of amino acids present in a particular protein sequence but absent from others. The accession numbers of the sequences used are Homo sapiens (PTBP1) (NP_002810.1); Mus musculus (PTBP1 (NP_001070831.1); Rattus norvegicus (PTBP1) (NP_001257986.1); Monodelphis domestica (PTBP1) (XP_001375584.2); Gallus gallus (PTBP1) (XP_015155407.1). Anolis carolinensis (PTBP1) (XP_008101639.2); Rhinatrema bivittatum (PTBP1) (XP_029469360.1); Megalops cyprinoides (PTBP1) (XP_036373891.1). Callorhinchus milii (PTBP1) (XP_007899197.1); Homo sapiens (PTBP2) (NP_001287914.1); Mus musculus (PTBP2) (NP_062423.1); Rattus norvegicus (PTBP2) (XP_006233298.1); Monodelphis domestica (PTBP2) (XP_007485072.1); Gallus gallus (PTBP2) (XP_004936631.4); Anolis carolinensis (PTBP2) (XP_008107427.1); Rhinatrema bivittatum (PTBP2) (XP_029474787.1); Megalops cyprinoides (PTBP2) (XP_036409945.1); Callorhinchus milii (PTBP2) (XP_042191263.1). [file mmc1.pdf]

```
                                                   N-terminal region                                         RRM1
Homo sapiens (PTBP1)           1   MD   GI      VPD-IAVGTKRGSDELFSTCVTNGPFIMSSNSASA   A---NGNDSKKFKGDSRSAG-VPSRVIHIRK   65
Mus musculus (PTBP1)           1   MD   GI      VPD-IAVGTKRGSDELFSTCVSNGPFIMSS-SASA   A---NGNDSKKFKGDNRSAG-VPSRVIHVRK   64
Rattus norvegicus (PTBP1)      1   MD   GI      VPD-IAVGTKRGSDELFSTCVSNGPFIMSS-SASA   A---NGNDSKKFKGDNRSTG-VPSRVIHVRK   64
Monodelphis domestica (PTBP1)  1   MD   GI      VPD-ITVGTKRGSDELFSTCVTNGPFIMSSNSASA   A---NGNDSKKFKGDNRSPG-VPSRVIHVRK   65
Gallus gallus (PTBP1)          1   MD   GI      VQD-ITVGTKRGSDELFSTCVTNGPFIMSSNASSA   A---NGNDSKKFKGDSRSAG-VPSRVIHVRK   65
Anolis carolinensis (PTBP1)    1   ME[6]GV      VTDlIAVGLKRGSDELFPSGIAYGPLTMSNSTPAT   A---NGNDNKKFKGD-RSPC-SPSRVLHLRK   71
Rhinatrema bivittatum (PTBP1)  1   MD   GI      VQD-IGVGTKRGSDELFSSCVTNGSFIMSSSAS-A   A---NGNDSKKFKGDSRSVG-VASRVIHVRQ   64
Megalops cyprinoides (PTBP1)   1   MD   GR[18]VHD-ITVGTKRGSDELFSSCVSNGPYIMS-NSSGA   A---NGNDSKKFKGDIRSPG-IPSRVIHVRK   82
Callorhinchus milii (PTBP1)    1   MD   GI      VQD-ITVGTKRGSDELLSACVTNGPFIMSNSTPSA   GeytNGNDSKKFKGDNRSAGiVPSKVIHIRK   69
Homo sapiens (PTBP2)           1   MD   GI      VTE-VAVGVKRGSDELLSGSVLSSPNSNMSSMVVT   A---NGNDSKKFKGEDKMDG-APSRVLHIRK   65
Mus musculus (PTBP2)           1   MD   GI      VTE-VAVGVKRGSDELLSGSVLSSPNSNMSGMVVT   A---NGNDSKKFKGEDKMDG-APSRVLHIRK   65
Rattus norvegicus (PTBP2)      1   MD   GI      VTE-VAVGVKRGSDELLSGSVLSSPNSNMSGMVVT   A---NGNDSKKFKGEDKMDG-APSRVLHIRK   65
Monodelphis domestica (PTBP2)  1   MD   GI      VTD-VAVGVKRGSDELLSGSVLSSPNSNMSSMVVT   A---NGNDSKKFKGEDKMDG-APSRVLHIRK   65
Gallus gallus (PTBP2)          1   MD   GI      VTD-VAVGVKRGSDELLSGSVLNSPNSNMSSMVVT   A---NGNDNKKFKSEDKMDG-SPSRVLHIRK   65
Anolis carolinensis (PTBP2)    1   MD   GI      VTD-VAVGVKRGSDELLSGSVLSSPTTNMSSIVVT   A---NGNDNKKFKGEDKMDG-APSRVLHIRK   65
Rhinatrema bivittatum (PTBP2)  1   MD   GI      VTD-VAVGVKRGSDELLSGGVLNGPNSNMSSIVVT   A---NGNDSKKFKGDDKMDG-TPSRVLHIRK   65
Megalops cyprinoides (PTBP2)   1   MD   GI      VSD-VAVGVKRGSDELLSGSLYNSPSS------VT   A---NGNDSKKLRVDDRMDS-PPSRVLHIRK   59
Callorhinchus milii (PTBP2)    1   MD   GI      LTD-VTVGLKRGSEEL-SSAVLNSPNSTMSSVVVT[8]A---NGNDSKKFKGDDKMIA-SPSRVLHIRK   72

                                                  RRM1                                        Linker 1
                              66   LPIDVTEGEVISLGLPFGKVTNLLMLKGKNQAFIEMNTEEAANTMVNYYTSVTPVLRGQPIYIQFSNHKELKTDSSPNQA   145
                              65   LPSDVTEGEVISLGLPFGKVTNLLMLKGKNQAFIEMNTEEAANTMVNYYTSVAPVLRGQPIYIQFSNHKELKTDSSPNQA   144
                              65   LPSDVTEGEVISLGLPFGKVTNLLMLKGKNQAFIEMSTEEAANTMVNYYTSVAPVLRGQPIYIQFSNHKELKTDSSPNQA   144
                              66   LPGDVTEAEVISLGLPFGKVTNLLMLKGKNQAFLEMNTEEASNTMVSYYTTVTPVLRSQPIYIQFSNHKELKTDNSPNQA   145
                              66   LPSDVTEAEVISLGLPFGKVTNLLMLKGKNQAFIEMNTEEAANTMVNYYTTVTPVLRSQPIYIQFSNHKELKTDNSPNQA   145
                              72   IPNDVTEAEVISLGLPFGRVTNLLMLKGKSQAFLEMASEEAAVTMVNYYTPITPHLRSQPVYIQYSNHRELKTDNLPNQA   151
                              65   LPSDVTEAEVISLGLPFGKVTNLLMLKGKNQAFIEMNTEEAANTMVSYYTTVTPVLRSQPIFIQFSNHKELKTDNSPNQA   144
                              83   LPNDINEAEVISLGLPFGKVTNLLMLKGKNQAFIEMNTEEAAQTMVSYYSSVTPVIRNHPIFMQFSNHKELKTDNSPNQV   162
                              70   LPNDITEAEVISLGLPFGKVTNLLMLKGKNQAFIEMNSEEAANTMVSYYSTVTPYLRNHPIYIQYSNHKELKTDNSPNQA   149
                              66   LPGEVTETEVIALGLPFGKVTNILMLKGKNQAFLELATEEAAITMVNYYSAVTPHLRNQPIYIQYSNHKELKTDNTLNQ-   144
                              66   LPGEVTETEVIALGLPFGKVTNILMLKGKNQAFLELATEEAAITMVNYYSAVTPHLRNQPIYIQYSNHKELKTDNTLNQ-   144
                              66   LPGEVTETEVIALGLPFGKVTNILMLKGKNQAFLELATEEAAITMVNYYSAVTPHLRNQPIYIQYSNHKELKTDNTLNQ-   144
                              66   LPGEVTETEVIALGLPFGKVTNILMLKGKNQAFLELATEESAITMVNYYSAVTPHLRNQPIYIQYSNHKELKTDNTLNQ-   144
                              66   LPGEVTETEVIALGLPFGKVTNILMLKGKNQAFLELATEEAAITMVNYYSAVTPHLRNQPIYIQYSNHKELKTDNTLNQ-   144
                              66   LPGEVTETEVIALGLPFGKVTNILMLKGKNQAFLELATEEAAITMVNYYSAVTPHLRNQPIYIQYSNHKELKTDNTLNQ-   144
                              66   LPNEVTETEVIALGLPFGKVTNILMLKGKNQAFLELASEEAAITMVNYYTTMTPHLRNQPIYIQYSNHKELKTDNTLNQ-   144
                              60   LPNEVAETEVIALGLPFGKVTNILMLKGKNQAFLELGTEEAAVTMVNYYTAVTPHVRNVPVFIQYSNHKELKTDNALNQ-   138
                              73   LPSEVMETEVIALGLPFGKVTNILMLKGKNQAFLELASEEAAVTMVNYYTAVTPHLRNQPIYIQYSNHKELKTDTALNQ-   151

                                               Linker 1                              RRM2
                             146   RAQAALQAVNSVQSGNLALAASAAAVDAGMAMAGQSPVLRIIVENLFYPVTLDVLHQIFSKFGTVLKIITFTKNNQFQAL   225
                             145   RAQAALQAVNSVQSGNLALAASAAAVDAGMAMAGQSPVLRIIVENLFYPVTLDVLHQIFSKFGTVLKIITFTKNNQFQAL   224
                             145   RAQAALQAVNSVQSGNLALAASAAAVDAGMAMAGQSPVLRIIVENLFYPVTLDVLHQIFSKFGTVLKIITFTKNNQFQAL   224
                             146   RAQAALQAVNSVQSGNLALSASAAAADAGMAVAGQSPVLRIIVENLFYPVTLDVLHQIFSKFGTVLKIITFTKNNQFQAL   225
                             146   RAQAALQAVNSVQSGNLALPAPAAAVDAGMAMAGQSPVLRIIVENLFYPVTLDVLHQIFSKFGTVLKIITFTKNNQFQAL   225
                             152   RTQAALQAVSAVQSGGLALTG--APATEGGLPPGQSSVLRIIVENLFYPVTLEVLYQIFSKFGTVLKIITFTKNNQFQAL   229
                             145   RAQAALQAVNSVQSGNIALSASAAAVDAGMAMAGQSPVLRVIVENLFYPVTLDVLHQIFSKFGTVLKIITFTKNNQFQAL   224
                             163   RAQAALQAVNAVQTGSMSM----ASVDAS-GMGSHSPVLRIIVENLFYPVTLDVLHQIFSKFGTVLKIITFTKNNQFQAL   237
                             150   RAHAALQAVTAVQSANMAITGTGVA-DTSVALTGQSPVLRIIVENLFYPVTLDVLHQIFSKFGSVMKIITFTKNNQFQAL   228
                             145   RAQAVLQAVTAVQTANT--PLSGTTVSESAVTPAQSPVLRIIIDNMYYPVTLDVLHQIFSKFGAVLKIITFTKNNQFQAL   222
                             145   RAQVVLQAVTAVQTANT--PLSGTTVSESAVTPAQSPVLRIIIDNMYYPVTLDVLHQIFSKFGAVLKIITFTKNNQFQAL   222
                             145   RAQVVLQAVTAVQTANT--PLSGTTVSESAVTPAQSPVLRIIIDNMYYPVTLDVLHQIFSKFGAVLKIITFTKNNQFQAL   222
                             145   RAQAVLQAVTAVQTANT--PLSGTTVSESAVTPAQSPVLRIIIDNMYYPVTLDVLHQIFSKFGAVLKIITFTKNNQFQAL   222
                             145   RAQAVLQAVTAVQATNA--PISGTTVSESAVTPAQSPVLRIIIDNMYYPVTLDVLHQIFSKFGAVLKIITFTKNNQFQAL   222
                             145   RAQAVLQAVTAVQTTNT--PISGTTVSESAVTPAQSPVLRIIIDNMYYPVTLDVLHQIFSKFGAVLKIITFTKNNQFQAL   222
                             145   RAQAVLQAVTAVQTSNS--SLSGTAVSESMATAAQSPVLRIIIDNMYYPVTLDVLHQIFSKFGAVLKIITFTKNNQFQAL   222
                             139   RAQAVLQAVSAVQAGGT--PTSGSTASESALTPAPSPVLRIIIDNMFYPVTLDVLQQIFSKFGTVMKIITFTKNNQFQAL   216
                             152   RAQAVLQAVTAVQTGGT--PLTGTTISENAVTPPQSPVLRIIIDNMYYPVTLDVLYQIFSKFGSVMKIITFXKNNQFQAL   229

                                                     RRM2                                 Linker 2
                             226   LQYADPVSAQHAKLSLDGQNIYNACCTLRIDFSKLTSLNVKYNNDKSRDYTRPDLPSGDSQPSLD-QTMAAAFGAP-GII   303
                             225   LQYADPVSAQHAKLSLDGQNIYNACCTLRIDFSKLTSLNVKYNNDKSRDYTRPDLPSGDSQPSLD-QTMAAAFGAP-GIM   302
                             225   LQYADPVSAQHAKLSLDGQNIYNACCTLRIDFSKLTSLNVKYNNDKSRDYTRPDLPSGDSQPSLD-QTMAAAFGAP-GIM   302
                             226   LQYSDPVSAQHAKLSLDGQNIYNACCTLRIDFSKLTSLNVKYNNDKSRDYTRPDLPSGDSQPSLD-QTMAAAFGAP-GII   303
                             226   LQYADPMSAQHAKLSLDGQNIYNACCTLRIDFSKLTSLNVKYNNDKSRDYTRPDLPSGDNQPPLD-QTMAAAFGAP-GII   303
                             230   LQYADPLNAHYARMALDGQNIYNACCTLRIDFSKLTSLNVKYNNDKSRDFTRLDLPSGDGQPSLE-PTMAAAFGTP-GII   307
                             225   LQYADPMSAQHAKLSLDGQNIYNACCTLRIDFSKLTSLNVKYNNDKSRDYTRPDLPSGDGQPSLD-QTM-AAFGAP-GII   301
                             238   LQYADGLTAQHAKLTLDGQNIYNACCTLRINFSKLTSLNVKYNNDKSRDYTRPDLPTGDSQPSIDhQAMAAAFGAP-GII   316
                             229   LQYTDGPSAQHAKLALDGQNIYNACCTLRIDFSKLTSLNVKYNNDKSRDYTRPDLPSGDSQPTLD-QTMAAAFGAP-GII   306
                             223   LQYGDPVNAQQAKLALDGQNIYNACCTLRIDFSKLVNLNVKYNNDKSRDYTRPDLPSGDGQPALD-PAIAAAFAKE-TSL   300
                             223   LQYGDPVNAQQAKLALDGQNIYNACCTLRIDFSKLVNLNVKYNNDKSRDYTRPDLPSGDGQPALD-PAIAAAFAKE-TSL   300
                             223   LQYGDPVNAQQAKLALDGQNIYNACCTLRIDFSKLVNLNVKYNNDKSRDYTRPDLPSGDGQPALD-PAIAAAFAKE-TSL   300
                             223   LQYGDPVNAQQAKLALDGQNIYNACCTLRIDFSKLVNLNVKYNNDKSRDYTRPDLPSGDGQPALD-PAIAAAFAKE-TSL   300
                             223   LQYGDPVNAQQAKLALDGQNIYNACCTLRIDFSKLVNLNVKYNNDKSRDYTRPDLPSGDGQPALD-PAIAAAFAKE-TSL   300
                             223   LQYGDPVNAQQAKLALDGQNIYNACCTLRIDFSKLVNLNVKYNNDKSRDYTRPDLPSGDGQPALD-PAIAAAFAKE-TSL   300
                             223   LQYGDPVNAQQAKLALDGQNIYNACCTLRIDFSKLVNLNVKYNNDKSRDYTRPDLPSGDGQPALD-PAIAAAFAKE-TSL   300
                             217   LQFSDPVNAQQAKLSLDGQNIYNACCTLRIDFSKLINLNVKYNNDKSRDYTRPELPAGDGQPAID-PSVAAALTKDsSSL   295
                             230   LQYSEPLNAQQAKLSLDGQNIYNACCTLRIDFSKLVNLNVKYNNDKSRDYTRPELPAGDGQPTMD-PAIAAAFAKE-NSL   307

                                                  Linker 2                                     RRM3
                             304   SaSPYAGA-GFPPTFAIPQAAGLSVPNVH     GALAPLAIP-S-AAAAAAAAGRIAIPGLAGA-GNSVLLVSNLNPERV   375
                             303   SaSPYAGA-GFPPTFAIPQAAGLSVPNVH     GALAPLAIP-S-AAAAAAAS-RIAIPGLAGA-GNSVLLVSNLNPERV   373
                             303   SaSPYAGA-GFPPTFAIPQAAGLSVPNVH     GALAPLAIP-S-AAAAAAAAGRIAIPGLAGA-GNSVLLVSNLNPERV   374
                             304   SaSPYAGA-GFPPTFAIPQAAGLSVPNVH     GALAPLAIP-S-AAAAAAAAGRIAIPGLTGA-GNSVLLVSNLNPERV   375
```

```
304  PaSPYAGA-GFPPTFAIPQAAGLTVQNVH    GALAPLAIP---AAAAAAAAGRIAIPGLAGA-GNSVLLVSNLNPERV  374
308  S-SPYAGAaGFAPAIGFPQAAGLSVQGVP    SALGPLAIT-T-STMT----GRMAIPGVHGMpGNSVLLVSNLNPDAI  376
302  SaSPYAGA-GFPPTFAIPQAAGLSVPGVP    GTLAPLAIP-SAAAAAAAAGRLGIPGLTGP-GNCVLLVSSLNPERV  374
317  SaTPYAGAhGFPPAFAIQQAAGLSMPGVP    GALASLAIP-G-AAAAAAAAGRLGFTGLTG--GHCVLLVSNLNPERV  388
307  S-SPYGGA-GFPPSIAFQQA-GLSVPGVH    -SLAPLGMP-S-AAAAAAAASRMGIPGFSSL-GNTVLLVSNLNPERV  375
301  --------------------LGLPVAAVP    GALSPLAIPnAAAAAAAAAAGRVGMPGVSAG-GNTVLLVSNLNEEMV  355
301  --------------------L-----AVP    GALSPLAIPnAAAAAAAAAAGRVGMPGVSAG-GNTVLLVSNLNEEMV  350
301  --------------------LGLPVAAVP    GALSPLAIPnAAAAAAAAAAGRVGMPGVSAG-GNTVLLVSNLNEEMV  355
301  --------------------LGLPVAAVP    GALSPLAIPnAAAAAAAAAAGRVGMPGVSAG-GNTVLLVSNLNEEMV  355
301  --------------------LGLPVAAVP    GALSPLAIPnAAAAAAAAAAGRVGMPGVSAS-GNTVLLVSNLNEEMV  355
301  --------------------LGLPVAAVP    GALSPLGIPnAAAAAAAAAAGRVGMPGVSSG-GNTVLLVSNLNEEMV  355
301  --------------------LGLPVAAVP    GALSPLGIPnAAAAAAAAAAGRVGIPGVSSG-GNTVLLVSNLNEEMV  355
296  --------------------LGTPSGMVA[18]GALSPL----SAAAAAAAAAGRVALSGHS-G-TSGVLLVSNLNEEMV  363
308  --------------------L-----AVP    GSLSPLGI-sGAAAAAAAAAGRVAMSGVGSG-NHSVLLVSNLNDEMV  356

                                    RRM3
376  TPQSLFILFGVYGDVQRVKILFNKKENALVQMADGNQAQLAMSHLNGHKLHGKPIRITLSKHQNVQLPREGQEDQGLTKD  455
374  TPQSLFILFGVYGDVQRVKILFNKKENALVQMADGSQAQLAMSHLNGHKLHGKSVRITLSKHQSVQLPREGQEDQGLTKD  453
375  TPQSLFILFGVYGDVQRVKILFNKKENALVQMADGSQAQLAMSHLNGHKLHGKSVRITLSKHQSVQLPREGQEDQGLTKD  454
376  TPQCLFILFGVYGDVQRVKILFNKKENALVQMADGNQAQLAMSHLNGQKLHGKPIRITLSKHQTVQLPREGQEDQGLTKD  455
375  TPQCLFILFGVYGDVQRVKILFNKKENALVQMADGNQAQLAMSHLNGQKLHGKPIRITLSKHQTVQLPREGQEDQGLTKD  454
377  TPDGLFILFGVYGDVHRVKIMFNKKENALVQMADATQAQLAMNHLNGQKLYGKMLRVTLSKHQTVQLPREGQEDQGLTKD  456
375  TPQCLFILFGVYGDVQRVKILFNKKENALVQMADGNQAQLAMSHLNGQRLHGKPIRIALSKHQTVQLPREGQEDQGLTKD  454
389  TPQCLFILFGVYGDVMRVKILFNKKENALVQMADGTQAQLAMSHLNGQKLHGKAIRVTLSKHTNVQLPREGQEDQGLTKD  468
376  TPKCLFILFGVYGDVHRVKILFNKKENSLVQMADSNQSQLAMSHLNGQRLHGKAMRVTMSKHQTVQLPREGQEDQGLTKD  455
356  TPQSLFTLFGVYGDVQRVKILYNKKDSALIQMADGNQSQLAMNHLNGQKMYGKIIRVTLSKHQTVQLPREGLDDQGLTKD  435
351  TPQSLFTLFGVYGDVQRVKILYNKKDSALIQMADGNQSQLAMNHLNGQKMYGKIIRVTLSKHQTVQLPREGLDDQGLTKD  430
356  TPQSLFTLFGVYGDVQRVKILYNKKDSALIQMADGNQSQLAMNHLNGQKMYGKIIRVTLSKHQTVQLPREGLDDQGLTKD  435
356  TPQSLFTLFGVYGDVQRVKILYNKKDSALIQMADGNQSQLAMNHLNGQKMYGKIIRVTLSKHQTVQLPREGLDDQGLTKD  435
356  TPQSLFTLFGVYGDVQRVKILYNKKDSALIQMADGNQSQLAMSHLNGQKMYGKIIRVTLSKHQTVQLPREGLDDQGLTKD  435
356  TPQSLFTLFGVYGDVQRVKILYNKKDSALIQMADGNQSQLAMSHLNGQKMYGKIIRVTLSKHQTVQLPREGLDDQGLTKD  435
356  SPQSLFTLFGVYGDVQRVKILYNKKDSALIQMADGNQSQLAMSHLNGQKMYGKIIRVTLSKHQTVQLPREGLDDQGLTKD  435
364  TPQSLFTLFGVYGDAQRVKILYNKKDSALIQMADGNQAKLAMSHLNGQKMYGKIIRVTLSKHQTVQLPREGLDDQGLTKD  443
357  TPQSLFTLFGVYGDVQRVKILYNKKDSALVQMADVNQAQLAMSHLNGQKMYGKIIRVTLSKHQTVQLPREGLDDQGLTKD  436

  RRM3       Linker 3                          RRM4
456  YGNSPLHRFKKPGSKNFQNIFPPSATLHLSNIPPSVSEEDLKVLFSSNGGVVKGFKFFQKDRKMALIQMGSVEEAVQALI  535
454  YGSSPLHRFKKPGSKNFQNIFPPSATLHLSNIPPSVSEDDLKSLFSSNGGVVKGFKFFQKDRKMALIQMGSVEEAVQALI  533
455  YGSSPLHRFKKPGSKNFQNIFPPSATLHLSNIPPSVSEDDLKSLFSSNGGVVKGFKFFQKDRKMALIQMGSVEEAVQALI  534
456  YGNSPLHRFKKPGSKNFQNIFPPSATLHLSNIPPSISEEDLKMLFSSNGGMVKGFKFFQKDRKMALIQMGSVEEAIQSLI  535
455  YGNSPLHRFKKPGSKNFQNIFPPSATLHLSNIPPSIAEEDLKMLFSSNGGMVKGFKFFQKDRKMALIQMGSVEEAIQSLI  534
457  YSNSPLHRFKKPGSKNFQNIFPPSATLHLSNIPPSVTVDDLKNLFADTGCIVKAFKFFPKDRKMALIQLGSVEEAIQALI  536
455  YSNSPLHRFKKPGSKNFQNIFPPSATLHLSNIPPSITEDDLKMLFSSNGGLVKGFKFFQKDHKMALIQMGSVEEAIQVLI  534
469  FSNSPLHRFKKPGSKNYQNIFPPSATLHLSNIPPSVVEDDLKMLFASSGALVKAFKFFQKDRKMALIQMGSVEEAIQSLI  548
456  YSSSPLHRFKKPGSKNFQNIFPPSATLHLSNIPPSVTEDDLKMLFSTTNGMVKGFKFFQKDRKMALIQMGSVEEAIQALI  535
436  FGNSPLHRFKKPGSKNFQNIFPPSATLHLSNIPPSVAEEDLRTLFANTGGTVKAFKFFQRDHKMALLQMATVEEAIQALI  515
431  FGNSPLHRFKKPGSKNFQNIFPPSATLHLSNIPPSVAEEDLRTLFANTGGTVKAFKFFQRDHKMALLQMATVEEAIQALI  510
436  FGNSPLHRFKKPGSKNFQNIFPPSATLHLSNIPPSVAEEDLRTLFANTGGTVKAFKFFQRDHKMALLQMATVEEAIQALI  515
436  FGNSPLHRFKKPGSKNFQNIFPPSATLHLSNIPPSVAEEDLRTLFANTGGTVKAFKFFQRDHKMALLQMATVEEAIQALI  515
436  FGNSPLHRFKKPGSKNFQNIFPPSATLHLSNIPPSVAEEDLRTLFANTGGTVKAFKFFQRDHKMALLQMSTVEEAIQALI  515
436  FGNSPLHRFKKPGSKNFQNIFPPSATLHLSNIPPSVSEDDLRMLFANTGGTVKAFKFFQRDHKMALLQMSTVEEAIQALI  515
436  FGNSPLHRFKKPGSKNFQNIFPPSATLHLSNIPPSVSEDDLRALFANTGGTVKAFKFFQRDHKMALLQMSTVEEATQALI  515
444  FTNSPLHRFKKPGSKNFQNIFPPSATLHLSNIPQDVTEEDLRLLFSNSGGTVKAFKFFQ-DHKMALLQMATVEEAIQALI  522
437  FTNSPLHRFKKPGSKNFQNIFPPSATLHLSNISPAIDEDDIKALFINTGGNVKGFKFFQ-DHKMALLQMCSVEEAIQALI  515

          RRM4
536  DLHNHDLGENHHLRVSFSKSTI  557
534  ELHNHDLGENHHLRVSFSKSTI  555
535  ELHNHDLGENHHLRVSFSKSTI  556
536  DLHNHDLGENHHLRVSFSKSTI  557
535  DLHNHDLGENHHLRVSFSKSTI  556
537  ELHNHDLGENHHLRVSFSKSTI  558
535  ELHNHDLGDNHHLRVSFSKSTI  556
549  EFHNHDLGENHHLRVSFSKSTI  570
536  DLHNHDLGENHHLRVSFSKSTI  557
516  DLHNYNLGENHHLRVSFSKSTI  537
511  DLHNYNLGENHHLRVSFSKSTI  532
516  DLHNYNLGENHHLRVSFSKSTI  537
516  DLHNYNLGENHHLRVSFSKSTI  537
516  DLHNYNLGENHHLRVSFSKSTI  537
516  DLHNYNLGENHHLRVSFSKSTI  537
516  DLHNYNLGDNHHLRVSFSKSTI  537
523  DLHNYNMGENHYLRVSFSKSTI  544
516  DLHNYDLGENHHLRVSFSKSTI  537
```
