# Supplementary figures and images for: Unstructured linker regions play a role in the differential splicing activities of paralogous RNA binding proteins PTBP1 and PTBP2
Source: J Biol Chem. 2024 Feb 8;300(3):105733. doi: 10.1016/j.jbc.2024.105733 (PMC10914480; doi:10.1016/j.jbc.2024.105733)

PTBP1

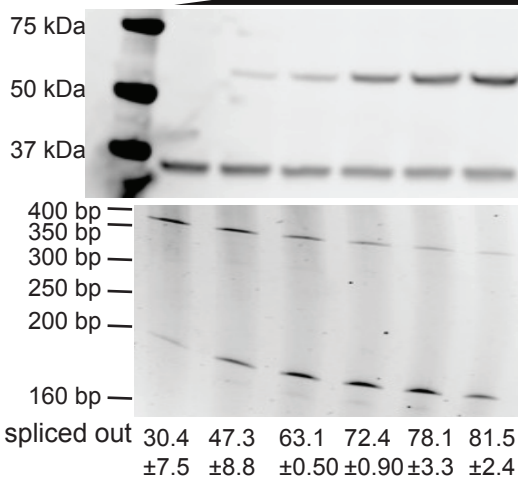

PTBP2

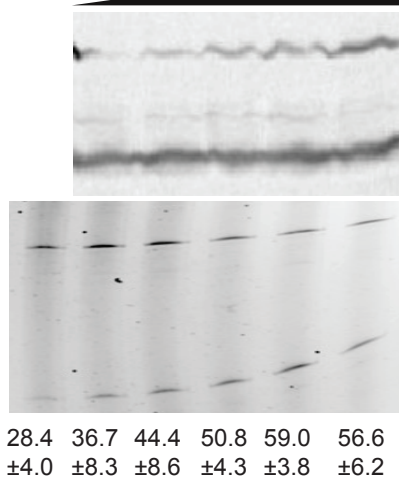

Chimera A

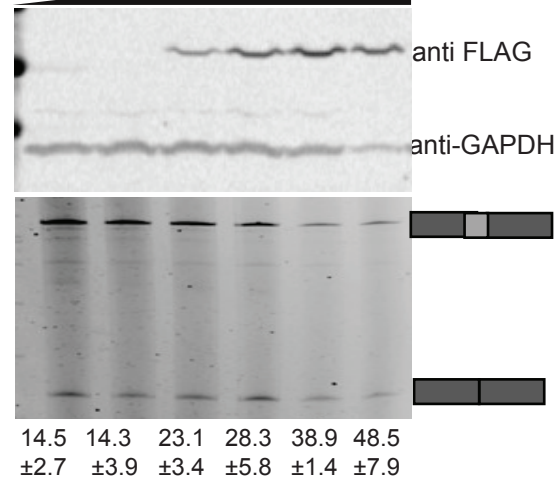

Chimera B

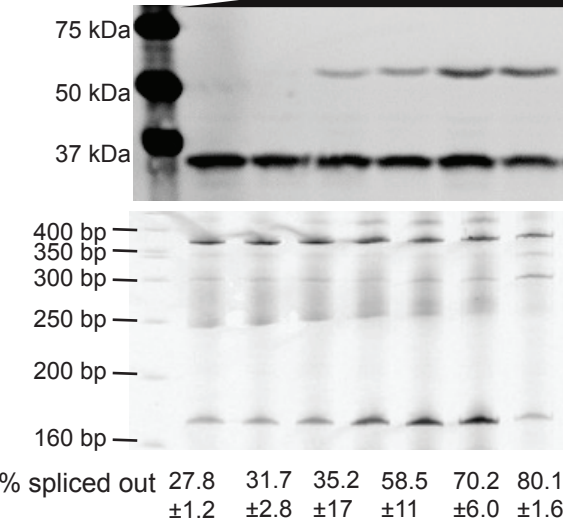

Chimera C

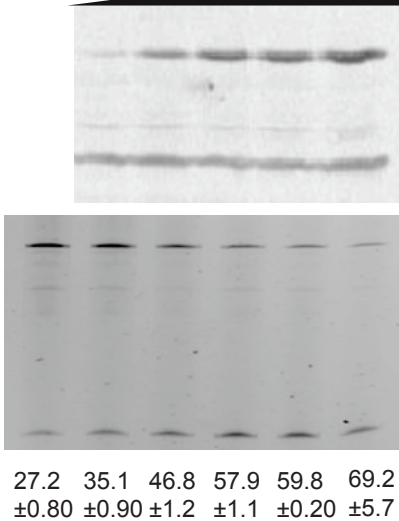

Chimera D

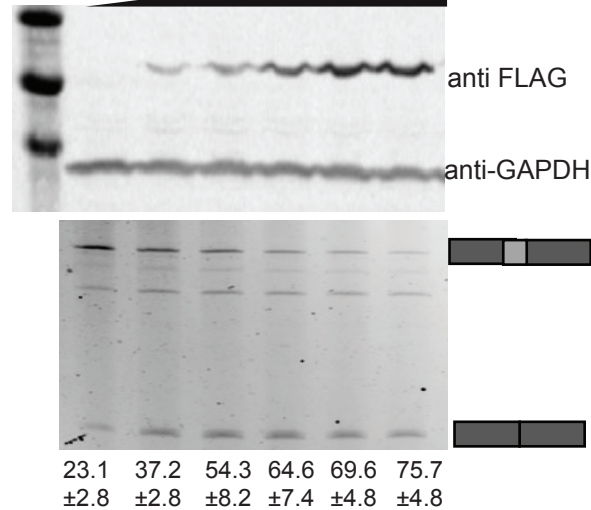

Chimera E

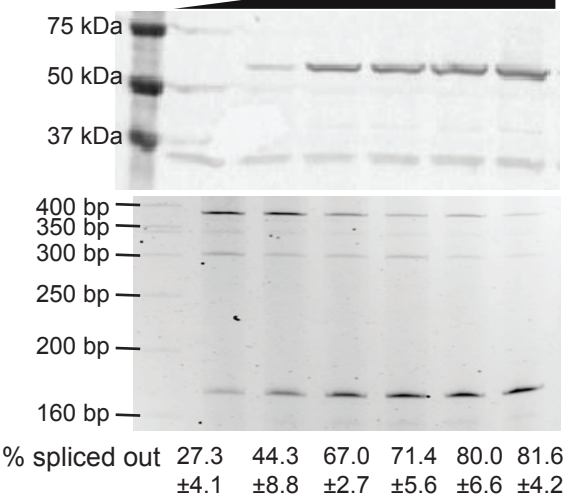

Chimera F

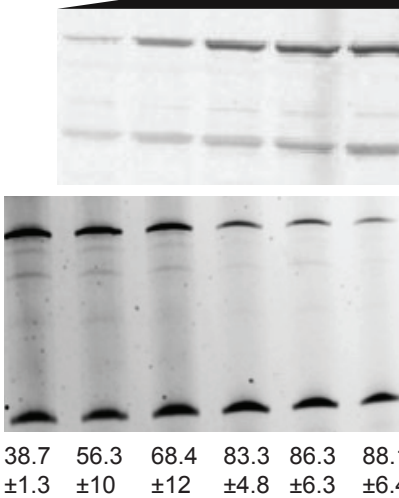

Chimera G

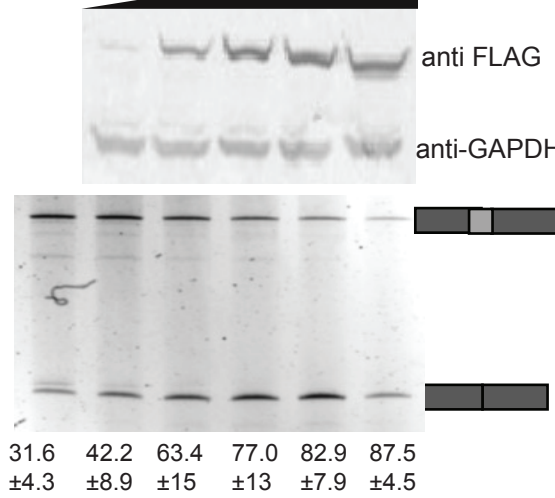

Supplement: Supporting Figure S2 — PTBP1, PTBP2 and chimera splicing activity on the Dup175 test exon. Each panel includes an immunoblot (top) and UREA-PAGE gel image (bottom) of protein expression and spliced products respectively of titrations of wild type and chimeric PTBP constructs. Immunoblots contain Flag-PTB proteins in cell lysates after transfection with increasing concentration of Flag-PTBP plasmid DNA (0.25, 0.5, 1.0, 1.5 and 2.0 ug). Molecular weight markers are indicated to the left of the first immunoblot in each panel. Immunoblots for PTBP2, Chimeras C, F and G do not contain the empty vector control sample lane. Splicing reporter minigene Dup 175-DS9 (0.5 μg) was cotransfected at each DNA concentration. RNA was harvested after 48 h, assayed by reverse transcription-PCR, and quantified. The level of percent-spliced-out was calculated by dividing band intensity for the excluded product by the total value of excluded and included products in each lane. The level of percent spliced out at each DNA concentration (0.25, 0.5, 1.0, 1.5, 2.0 ug) is calculated and indicated below each lane. Std.Deviations for wild type PTB proteins and mean error for chimeric constructs are indicated. [file mmc2.pdf]

Sppl Fig. 3

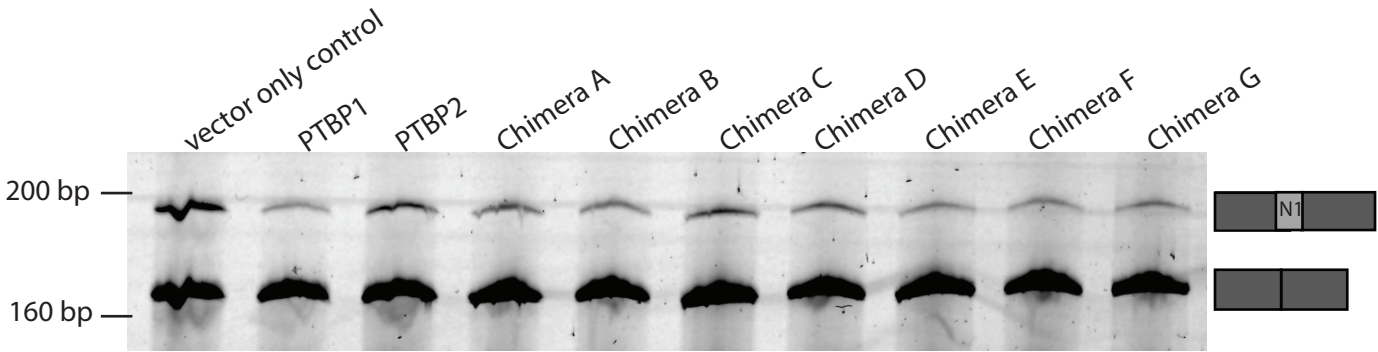

Supplement: Supporting Figure S3 — PTBP1, PTBP2 and chimera splicing activity on the neuronal N1 exon. Splicing reporter minigene Dup 4 to 5 (0.5 μg) was cotransfected with each FLAG construct into N2A cells. RNA was harvested after 48 h, assayed by reverse transcription-PCR using a fluorescent labeled reverse primer (5Alex488N/AACAGCATCAGGAGTGGACAGATCCC) and separated on a 7.5 M Urea 8% Acrylamide gel. N1 exon included and excluded band intensities were quantified using Image quant software. The level of percent-spliced-in was calculated by dividing band intensity for the included product by the total value of excluded and included products in each lane. [file mmc3.pdf]

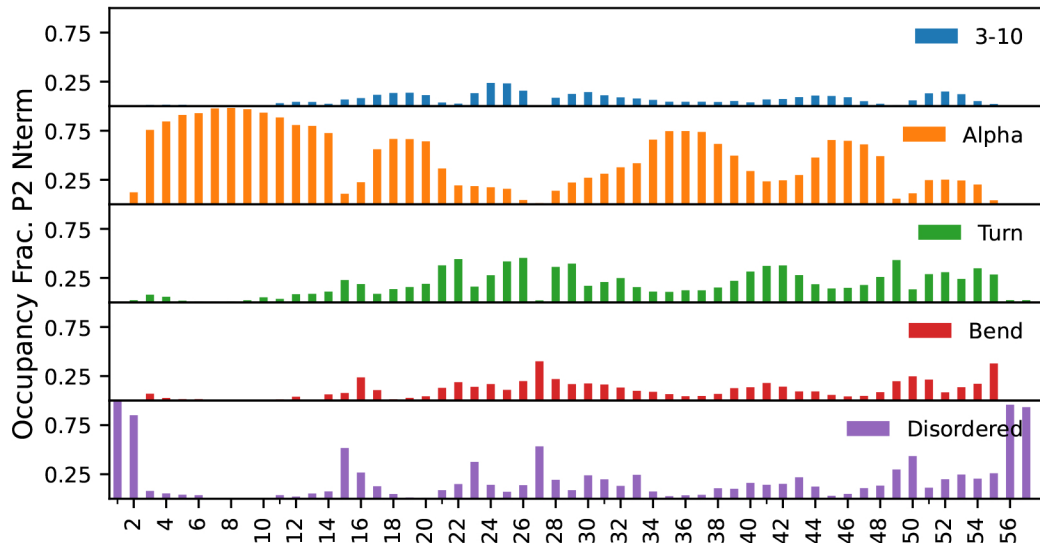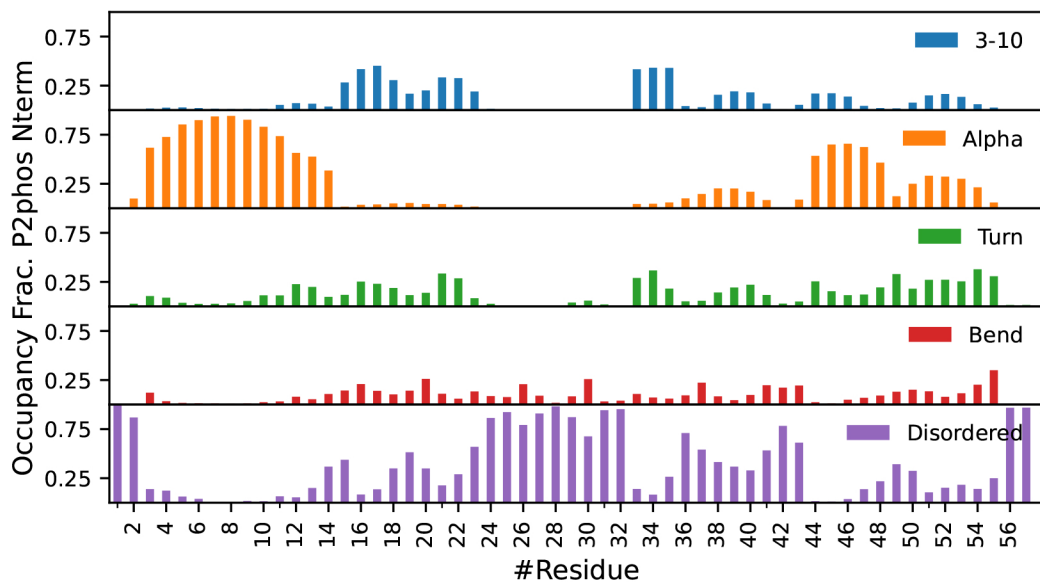

Supplement: Supporting Figure S4 — Secondary structure fractional occupancy of PTBP2’s N-terminal region from MD simulations: (top) P2Nterm, (bottom) P2phosNterm. [file mmc4.pdf]

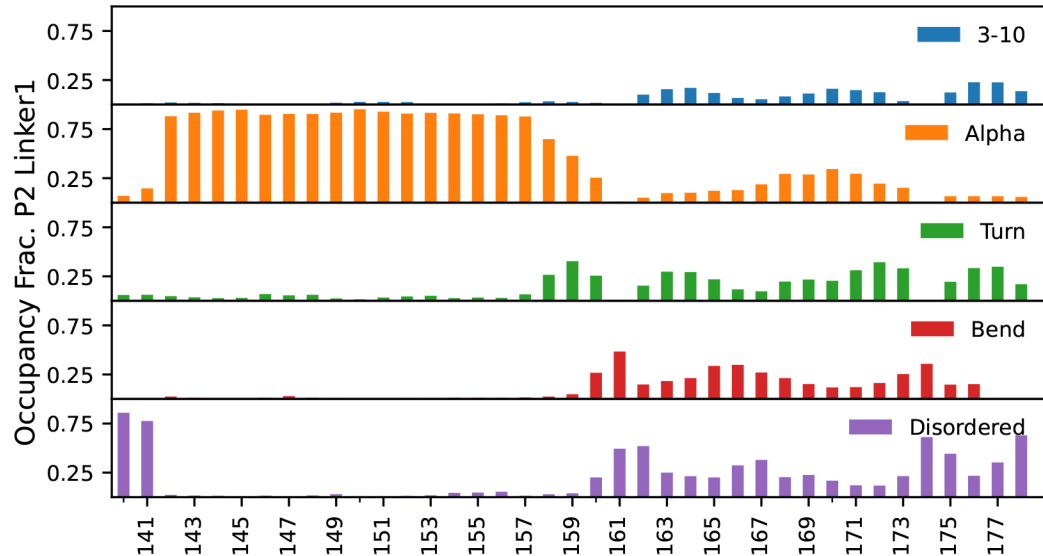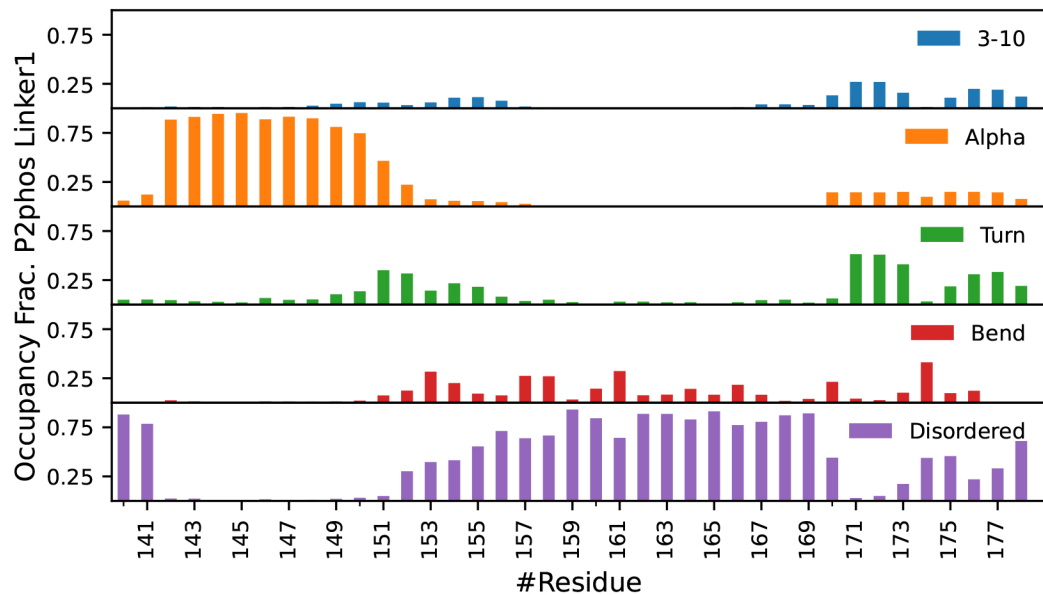

Supplement: Supporting Figure S5 — Secondary structure fractional occupancy of PTBP2’s Linker 1 region from MD simulations: (top) P2L1, (bottom) P2phosL1. [file mmc5.pdf]

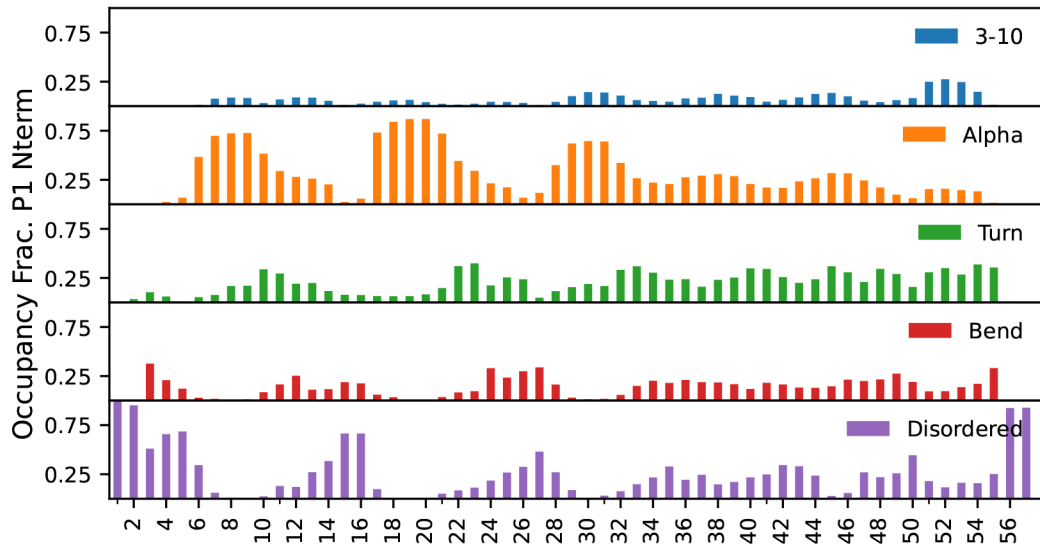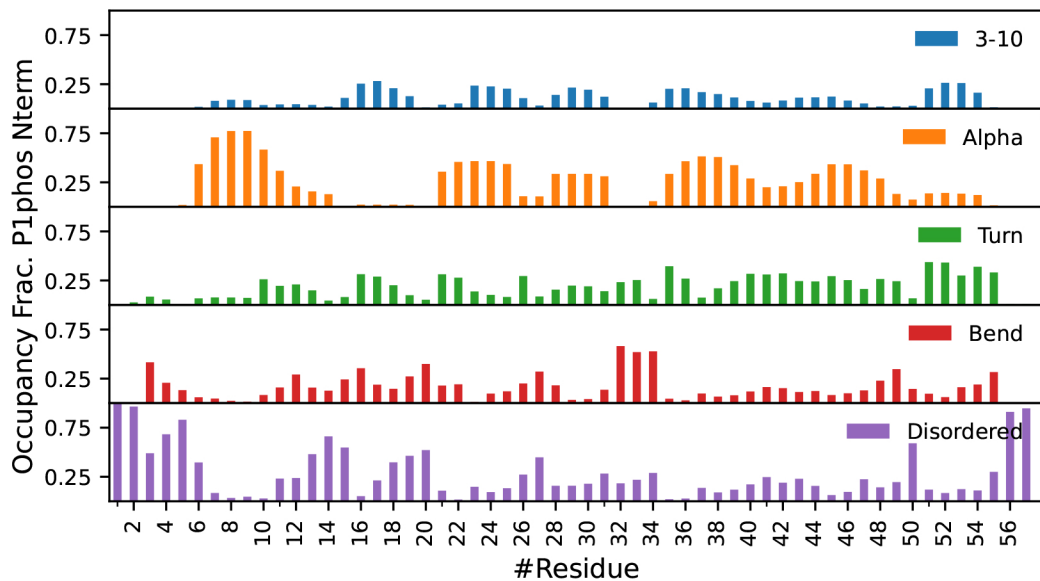

Supplement: Supporting Figure S6 — Secondary structure fractional occupancy of PTBP1’s N-terminal region from MD simulations: (top) P1Nterm, (bottom) P1phosNterm. [file mmc6.pdf]

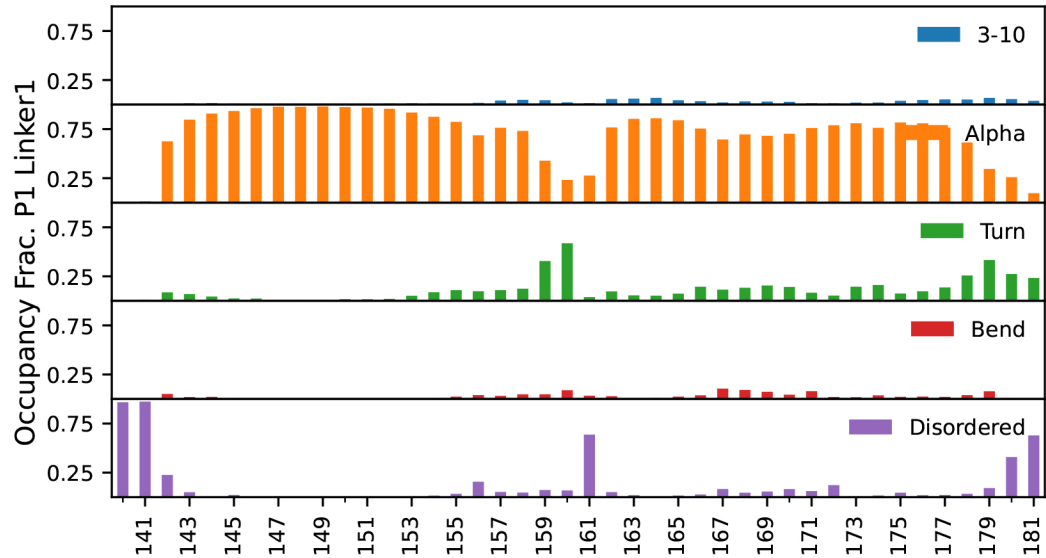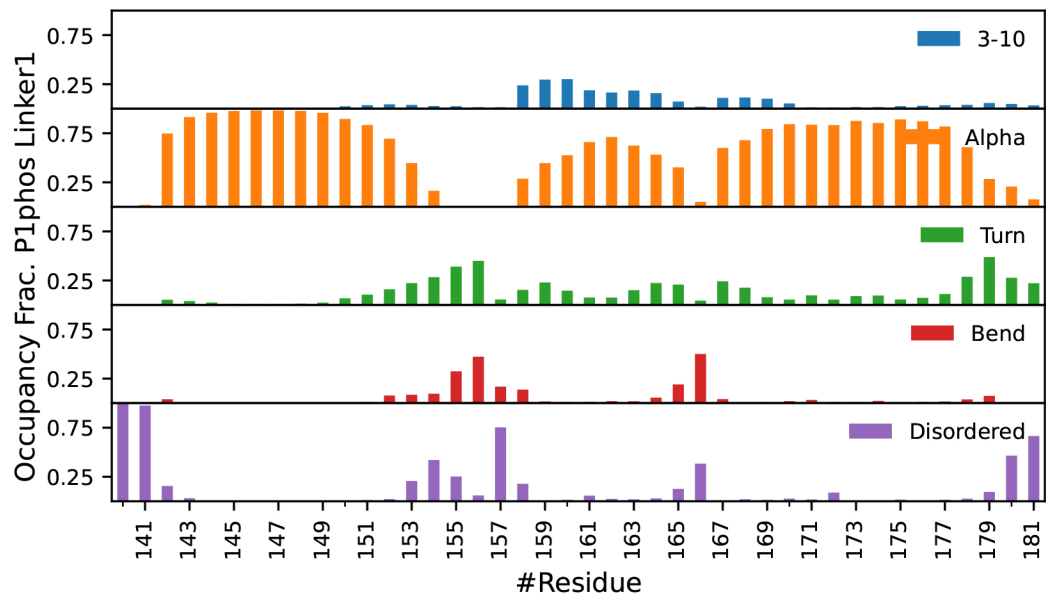

Supplement: Supporting Figure S7 — Secondary structure fractional occupancy of PTBP1’s Linker 1 region from MD simulations: (top) P1L1, (bottom) P1phosL1. [file mmc7.pdf]

Occupancy Frac. P1 Linker2

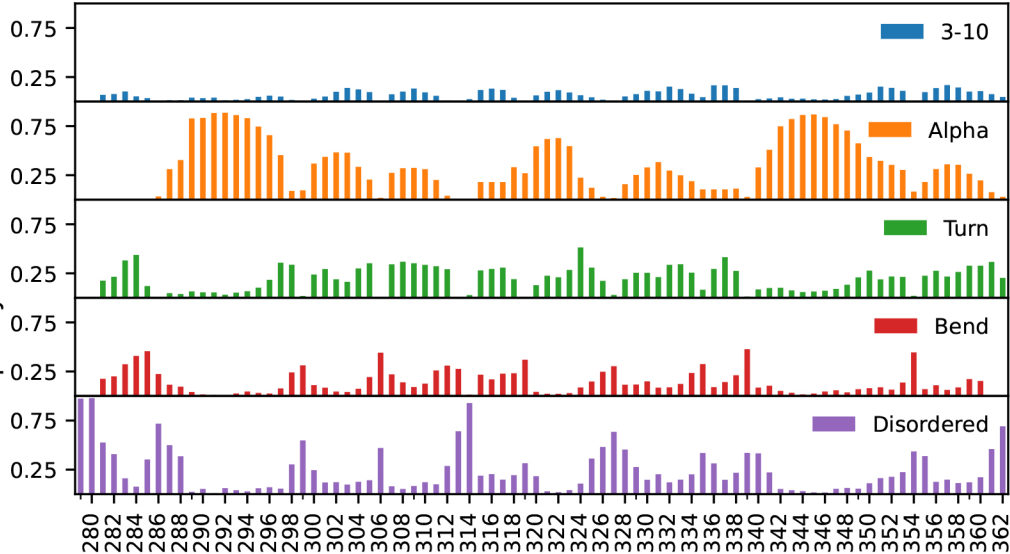

Occupancy Frac. P1phos Linker2

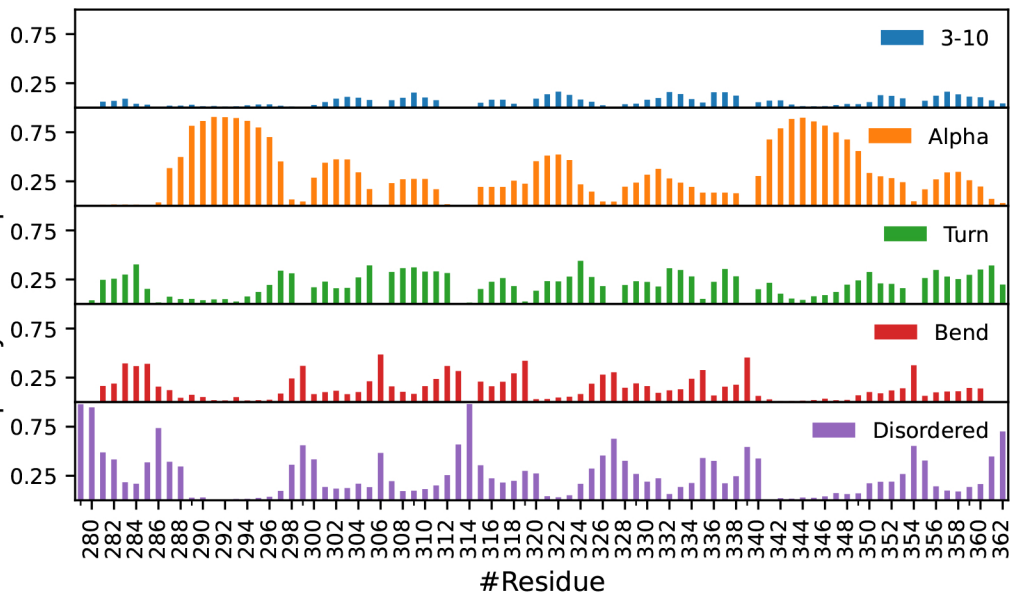

#Residue

Supplement: Supporting Figure S8 — Secondary structure fractional occupancy of PTBP1’s Linker 2 region from MD simulations: (top) P1L2, (bottom) P1phosL2. [file mmc8.pdf]

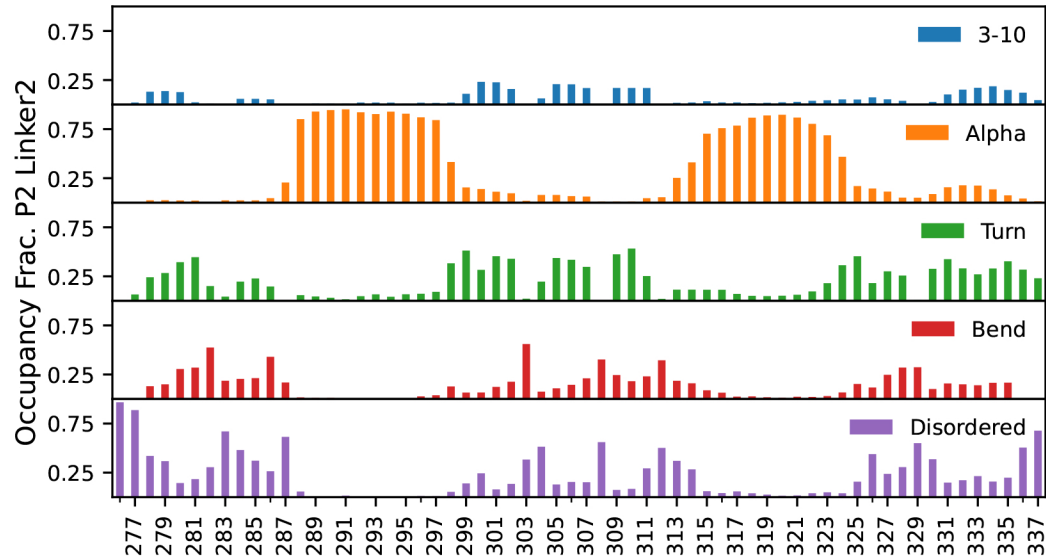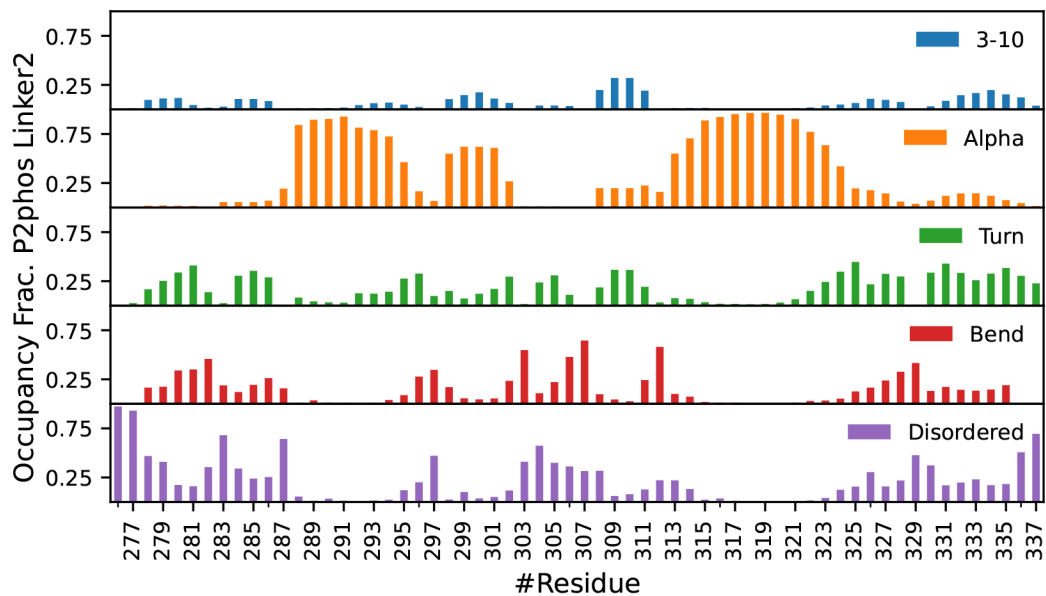

Supplement: Supporting Figure S9 — Secondary structure fractional occupancy of PTBP2’s Linker 2 region from MD simulations: (top) P2L2, (bottom) P2phosL2. [file mmc9.pdf]
