# Supplementary material for: Unstructured linker regions play a role in the differential splicing activities of paralogous RNA binding proteins PTBP1 and PTBP2
Source: J Biol Chem. 2024 Feb 8;300(3):105733. doi: 10.1016/j.jbc.2024.105733 (PMC10914480; doi:10.1016/j.jbc.2024.105733)
Supplement: Supporting Table S1 [file mmc10.docx]

**Sppl. Table 1.** Temperatures used in TREMD simulations.

| Sequence | Temperatures |
| --- | --- |
| P1L1  P1L2 | 275.00, 287.47, 298, 300.44, 313.91, 327.91, 342.46, 357.65, 373.38, 389.72, 406.71, 424.35, 442.69, 461.75, 481.55, 500.00  275.00, 283.86, 292.96, 298, 302.31, 311.93, 321.81, 331.98, 342.43, 353.17, 364.21, 375.56, 387.22, 399.21, 411.53, 424.20, 437.22, 450.60, 464.48, 478.61, 493.16, 500.00 |
| P1Nterm  P2L1  P2L2  P2Nterm | 275.00, 285.42, 298, 307.15, 318.61, 330.45, 342.68, 355.31, 368.35, 381.83, 395.75, 410.11, 424.94, 440.27, 456.09, 472.43, 489.24, 500.00  275.00, 287.47, 298, 300.44, 313.91, 327.91, 342.46, 357.65, 373.38, 389.72, 406.71, 424.35, 442.69, 461.75, 481.55, 500.00  275.00, 285.40, 298, 307.22, 318.67, 330.50, 342.74, 355.36, 368.40, 381.86, 395.76, 410.12, 424.95, 440.23, 456.04, 472.36, 489.21, 500.00  275.00, 285.53, 298, 307.72, 319.37, 331.41, 343.85, 356.71, 370.00, 383.72, 397.91, 412.56, 427.70, 443.33, 459.50, 476.20, 493.32, 500.00 |
