# Supplementary material for: Unstructured linker regions play a role in the differential splicing activities of paralogous RNA binding proteins PTBP1 and PTBP2
Source: J Biol Chem. 2024 Feb 8;300(3):105733. doi: 10.1016/j.jbc.2024.105733 (PMC10914480; doi:10.1016/j.jbc.2024.105733)
Supplement: Supporting Table S2 [file mmc11.docx]

**Sppl. Table 2**

Primer sequences for chimera construction. All sequences read 5’ to 3’. Primers were purchased to generate Chimeras A, B and C and the combination of forward and reverse primer in the first PCR reaction is indicated in parenthesis. The resulting fragments and combinations were used to generate Chimeras D, E, F and G.

| **Chimera A forward** (with PTBP2 reverse primer in 1^st^ PCR reaction)  CGAAGTGCAGGCGTCCCCAGTCGAGTTCTTCACATCAGAAAGC |
| --- |
| **Chimera A reverse** (with pcDNA3.1+ forward primer in 1^st^ PCR reaction)  GCTTTCTGATGTGAAGAACTCGACTGGGGACGCCTGCACTTCG |
| **Chimera B1 forward** (with B2 reverse primer in 1^st^ PCR reaction)  CCATAAGGAGCTTAAGACTGACAGCTCTCCCAACCAGGCGCGG |
| **Chimera B1 reverse** (with pcDNA3.1+ primer in 1^st^ PCR reaction)  CCATAAGGAGCTTAAGACTGACAGCTCTCCCAACCAGGCGCGG |
| **Chimera B2 forward** (with PTBP2 reverse primer in 1^st^ PCR reaction)  GGGATGGCGATGGCCGGGCAGAGCCCAGTATTGCGAATCATCAT |
| **Chimera B2 reverse** (with B1 forward primer in 1^st^ PCR reaction)  ATGATGATTCGCAATACTGGGCTCTGCCCGGCCATCGCCATCCC |
| **Chimera C1 forward** (with C2 reverse primer in 1^st^ PCR reaction)  GTAGAGATTATACTCGCCCGGACCTGCCTTCCGGGGACAGCC |
| **Chimera C1 reverse** (with pcDNA3.1+ primer in 1^st^ PCR reaction)  GGCTGTCCCCGGAAGGCAGGTCCGGGCGAGTATAATCTCTAC |
| **Chimera C2 forward** (with PTBP2 reverse primer in 1^st^ PCR reaction)  CCTGGCGGGGGCAGGAAATACCGTCCTTCTTGTCTCAAATCT |
| **Chimera C2 reverse** (with C1 forward primer in 1^st^ PCR reaction)  AGATTTGAGACAAGAAGGACGGTATTTCCTGCCCCCGCCAGG |
| **pcDNA3.1+ forward primer**  GGAATTGCCCTTGGGATCCTTACCATGGACTACAAGG |
| **PTBP2 reverse primer**  TCTAGAAAGGATATCTCATTAAATGGTGGACTTGCT |
